# A General and Predictive Understanding of Thermal Transport from 1D- and 2D-Confined Nanostructures: Theory and Experiment

**DOI:** 10.1021/acsnano.1c01946

**Published:** 2021-07-30

**Authors:** Albert Beardo, Joshua L. Knobloch, Lluc Sendra, Javier Bafaluy, Travis D. Frazer, Weilun Chao, Jorge N. Hernandez-Charpak, Henry C. Kapteyn, Begoña Abad, Margaret M. Murnane, F. Xavier Alvarez, Juan Camacho

**Affiliations:** † Physics Department, Universitat Autònoma de Barcelona, Bellaterra, Catalonia 08193, Spain; ‡ Department of Physics, JILA, and STROBE NSF Science & Technology Center, University of Colorado and NIST, Boulder, Colorado 80309, United States; § Center for X-Ray Optics, Lawrence Berkeley National Laboratory, Berkeley, California 94720, United States

**Keywords:** phonon hydrodynamics, non-Fourier heat transport, silicon, high-order harmonic generation, pump−probe spectroscopy

## Abstract

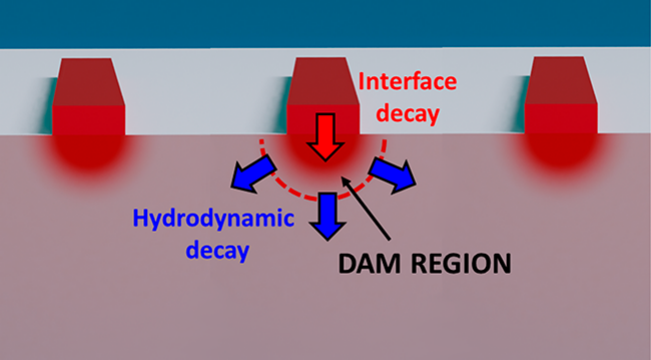

Heat management is crucial in the design of nanoscale devices as the operating temperature determines their efficiency and lifetime. Past experimental and theoretical works exploring nanoscale heat transport in semiconductors addressed known deviations from Fourier’s law modeling by including *effective* parameters, such as a size-dependent thermal conductivity. However, recent experiments have qualitatively shown behavior that cannot be modeled in this way. Here, we combine advanced experiment and theory to show that the cooling of 1D- and 2D-confined nanoscale hot spots on silicon can be described using a general hydrodynamic heat transport model, contrary to previous understanding of heat flow in bulk silicon. We use a comprehensive set of extreme ultraviolet scatterometry measurements of nondiffusive transport from transiently heated nanolines and nanodots to validate and generalize our *ab initio* model, that does not need any geometry-dependent fitting parameters. This allows us to uncover the existence of two distinct time scales and heat transport mechanisms: an interface resistance regime that dominates on short time scales and a hydrodynamic-like phonon transport regime that dominates on longer time scales. Moreover, our model can predict the full thermomechanical response on nanometer length scales and picosecond time scales for arbitrary geometries, providing an advanced practical tool for thermal management of nanoscale technologies. Furthermore, we derive analytical expressions for the transport time scales, valid for a subset of geometries, supplying a route for optimizing heat dissipation.

Advances in fabrication have scaled the characteristic dimensions of complex systems to the few nanometer range and even thinner. At these length scales, conventional macroscopic (bulk) models can fail to accurately describe nanoscale behavior because of the dominance of interfaces and surfaces. Specifically, thermal transport from nanoscale heat sources on semiconductor substrates strongly deviates from bulk diffusive transport predictions. Experiments show that as the heat source size is reduced, the heat transport efficiency falls well below what is predicted by bulk diffusion, both for structured optical excitation^1−4^ or for optically excited nanostructured transducers.^5−12^ Moreover, recent experiments have uncovered that both the size and spacing of periodic nanoheater arrays strongly influence thermal transport, resulting in counterintuitive behaviors.^8,9^ However, there is still no consensus on the underlying physics—in large part because there is no comprehensive model to describe these new nanoscale thermal transport regimes. This precludes smart design for good thermal management in next-generation nanodevices.

Theoretical proposals based on truncated Levy flights,^13^ suppression of phonons,^8,14^ or relaxons^15^ have explained certain aspects of nondiffusive thermal transport for specific geometries. Phonon hydrodynamics^16−24^ has been also successfully used to explain thermal transport behavior on 2D materials,^25^ such as graphene,^26^ and even in bulk materials at very low temperatures.^27^ As this behavior is known to occur when “normal” phonon scattering events (i.e., processes that conserve quasi-momentum) dominate over “resistive” ones, the existence of hydrodynamic transport in bulk semiconductors, like silicon, at room temperature has been explicitly discarded.^28^

It is widely accepted that solving the Boltzmann transport equation with *ab initio* calculated parameters is the most precise way to describe the transport of phonons, which are the dominant heat carriers for semiconductor and dielectric materials.^29−31^ However, several difficulties associated with this approach limit its use at a practical level. First, this equation is challenging to solve in general because of the complexity of phonon collisions. To overcome this challenge, the relaxation time approximation is often used to simplify the collision expression.^13,32^ However, this approximation does not guarantee energy conservation, which can lead to invalid results.^13,33^ Second, complex geometries are challenging because one must model how each phonon mode interacts with every boundary present. Finally, coupling this equation to other phenomena, such as thermoelectricity or thermoelasticity, exponentially increases the computational requirements. These challenges can prevent microscopic models, like the Boltzmann transport equation, from being directly compared to experimental data.

Because microscopic models cannot be applied in many complex geometries, experiments often use an intermediate layer, or mesoscopic models, to compare results and theory. Most mesoscopic models to date are based on Fourier’s law of heat diffusion with the addition of phenomenological effective parameters. This approach fits effective parameters to experiments and then formulates theoretical models to connect the fitted values to *ab initio* calculations. Recent works have used this effective Fourier model to analyze heat dissipation away from metallic nanostructures of varying size and spacing.^5−9,11^ This can quantify the deviation from the diffusive prediction by fitting either an effective thermal boundary resistance between the transducer and substrate^7−9^ or an effective thermal conductivity of the substrate.^5,6,12,34−36^ These techniques have significantly advanced our understanding, making it possible to develop new experimental mean free path spectroscopy techniques,^1^ as well as uncovering new transport regimes dominated by the heat source spacing.^7,8^ However, using Fourier’s law as a mesoscopic model, even with effective parameters, can obscure the underlying physics and fails to predict thermal transport observed for all time and length scales.^19,22^ Most importantly, this approach is difficult to generalize to arbitrary geometries or materials.

In this work, we present a comprehensive set of dynamic EUV scatterometry measurements of nondiffusive heat flow away from 1D- and 2D-confined nanostructures on bulk silicon. We use this data to validate and generalize the Kinetic Collective Model (KCM),^37,38^ which is a mesoscopic model which uses a hydrodynamic-like heat transport equation^16^ with *ab initio* parameters. Contrary to conventional understanding, we show that heat transport away from nanoscale sources on bulk silicon can be predicted by the hydrodynamic equation. This generalizes the hydrodynamic framework to situations where phonon momentum is conserved, which applies not only when normal collisions dominate but in regions with size comparable to the average resistive phonon mean free path near heat sources and system boundaries.^22,38,39^ We also experimentally observe that closely spaced 2D-confined (nanodots) on a bulk silicon substrate cool faster than widely spaced ones, and that this effect is larger in 2D-confined than in 1D-confined (nanoline) sources observed by previous works.^8,9^ Moreover, we demonstrate that KCM both fully predicts the heat transport over a wide range of length-scales and time-scales from 1D- and 2D-confined heat sources on a silicon substrate—including the counterintuitive behavior of the closely spaced geometry—and captures the full thermomechanical response to the system, which is beyond the capabilities of microscopic models.

Our mesoscopic hydrodynamic model also provides insight into the fundamental transport behavior. KCM allows us to identify the time scales over which two different transport mechanisms are dominant: one characteristic time dominated by the thermal boundary resistance and another regime that is dominated by hydrodynamic heat transport. The latter mechanism is responsible for the slow thermal decay of small heat sources, and consequently, its reduction is responsible for the increased dissipation of close-packed nanoheaters. Furthermore, we develop a two-box model, derived from the hydrodynamic equation, which provides a physical interpretation and specific expressions for the two characteristic dissipation mechanisms. We confirm these findings by comparing our models to both past 1D-confined and new 1D- and 2D-confined experimental data. We conclude that KCM—involving only a few parameters—provides a predictive description of the thermal and mechanical response in these complex systems with highly nondiffusive behavior and has specific advantages over the traditional effective Fourier model. This work thus represents a significant advance in both experimental and modeling capabilities opening the door to improved thermal management in iterative nanoscale device design, including possible routes to increase clock rates in nanoelectronics by surpassing what has been called the “thermal wall”.^40,41^

We measure the heat dissipation away from 1D-confined periodic nanoline heat sources on a silicon substrate using dynamic EUV scatterometry similar to that of refs (7−9) but with significantly improved signal-to-noise ratio—by nearly 2 orders of magnitude. These improvements allow us to perform new measurements on 2D-confined periodic nanodot heat sources on a silicon substrate, which are more challenging than 1D measurements due the reduced fraction of surface covered by the heat sources. Both the nanodot and nanoline arrays were fabricated under identical conditions. Our time-resolved measurements use an ultrafast infrared pump laser pulse to rapidly excite thermal heating and expansion in the metallic structures. The resulting thermal and elastic surface deformation is monitored by measuring the change in diffraction efficiency of an ultrashort EUV probe pulse, as depicted in Figure 1 (see Methods). Using this technique, we observe the heat dissipation from nanodot arrays in general geometries without complex fabrication and from nanoline arrays down to 20 nm in size (*L*) and 80 nm in spacing (*P*).

**Figure 1:**
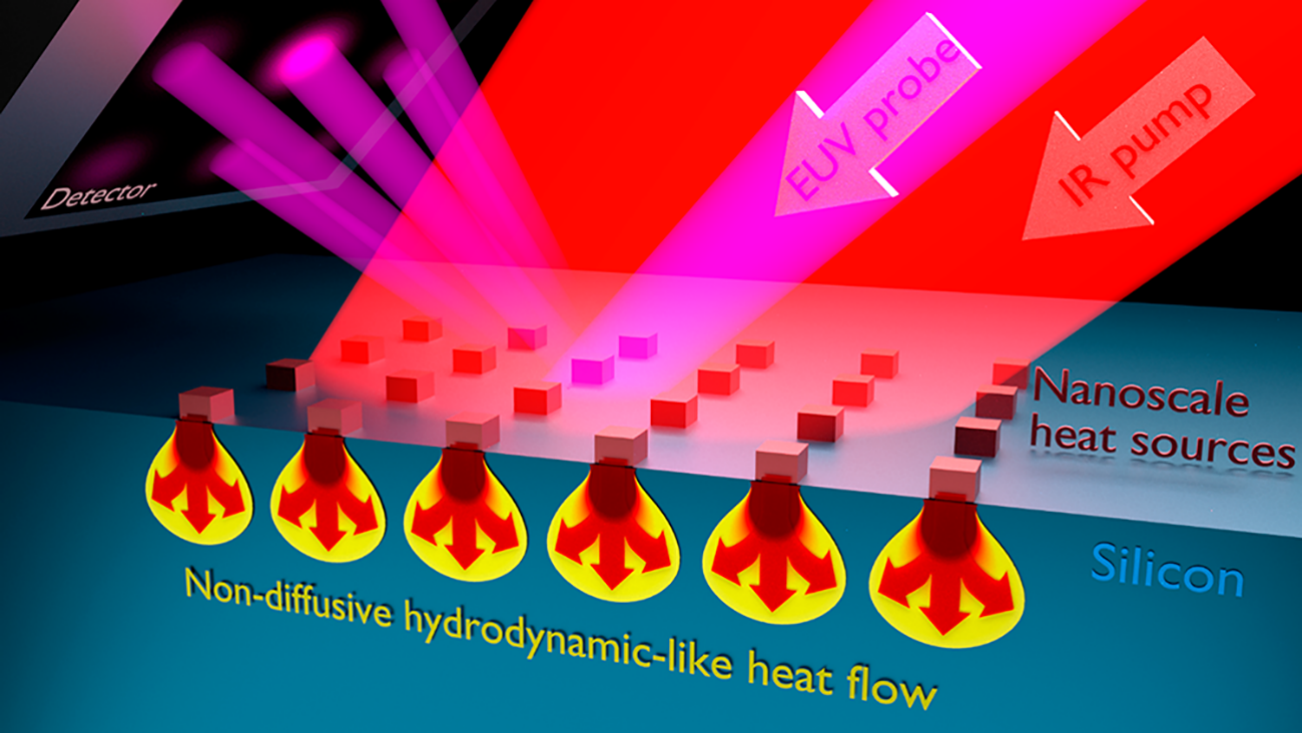
Schematic of dynamic EUV scatterometry for probing nondiffusive hydrodynamic-like heat flow. An ultrafast laser pulse rapidly heats the nanostructured transducers, which dissipate the thermal energy by transferring heat to the substrate. The heat flows away from the nanoscale heat sources following a nondiffusive hydrodynamic-like behavior, creating a “balloon” shaped temperature profile. The resulting surface deformation of the heated nanostructures and substrate is measured via diffraction of an ultrafast Extreme Ultraviolet (EUV) probe pulse, after a controlled pump–probe time delay. The EUV pulse scatters from the periodic nanostructure arrays into a detector. We reduce the recorded scattering pattern into a single value of diffraction efficiency as a function of time delay between the pump and probe pulses, which precisely tracks the thermal and elastic dynamics in the sample.

To interpret the experimental data, we implement a mesoscopic model using KCM and a thermoelastic set of equations (see Methods). For heat transport, we use Fourier’s law for the metal sources (which is dominated by electrons), and the Guyer and Krumhansl transport equation^16^ for the substrate (silicon), which is the material where non-Fourier behavior is expected:
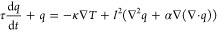
1where κ is the bulk thermal conductivity of substrate, τ the relaxation time of flux *q*, and *l* the nonlocal length—that can be microscopically interpreted as a weighted average phonon mean free path. All these parameters are intrinsic properties of the substrate. Equation (1) resembles the hydrodynamic Navier–Stokes equation of fluids; thus, we can build analogies between heat flow and fluid behavior. Fourier’s law can be easily recovered from eq 1: when the experiment time scales are much larger than τ, the first term can be neglected, and if spatial scales are much larger than *l*, the last term can be neglected. For sizes comparable to *l*, however, these viscous terms become important and capture the nondiffusive transport due to momentum conservation at the scale of the phonon mean free paths. In nanoscale regions near the heat sources, the momentum of emitted phonons is conserved due to the lack of resistive collisions. Hence, hydrodynamic effects can locally alter the heat transport even in semiconductors like silicon. In the limit where normal collisions dominate, Guyer and Krumhansl^16^ found α = 2; however, we use α = 1/3—analogous to a fluid with zero volume viscosity—in agreement with more recent works.^22,39,42,43^ To solve this equation, appropriate boundary conditions are implemented (see Methods and Supplementary Section 1). In addition, we require the thermal boundary resistance between the metal and the substrate. This boundary resistance is the only parameter in the model that cannot be derived from *ab initio* calculations since it is highly dependent on the fabrication process rather than being an intrinsic material property. However, as all our nanograting arrays have been fabricated in identical conditions, we use the identical value of the thermal boundary resistance for the entire data set. Given the *ab initio* values for the other parameters, the model can be solved by using finite elements to determine the evolution of the displacement, the temperature, and the heat flux in the nanostructure and substrate.^21^

The predictability of eq 1 has been recently validated in compact and holey films, and thermoreflectance experiments in silicon, with excellent agreement.^21,22,44^ As discussed in ref.,^21^ the applicability of the model with *ab initio* parameters (κ, *l*, and τ) is restricted to geometries where edge effects produced by two different boundaries do not overlap, i.e. when boundaries are separated by a distance larger than 2*l*. Here, the distance between heaters is *P* – *L* (see Figure 2). Thus, eq 1 is expected to be valid for nanostructure arrays satisfying *P* – *L* > 2*l*. We term experiments under this condition, where heaters are expected to behave independently, as effectively isolated heat sources, and those with *P* – *L* < 2*l* as close-packed heat sources.

**Figure 2:**
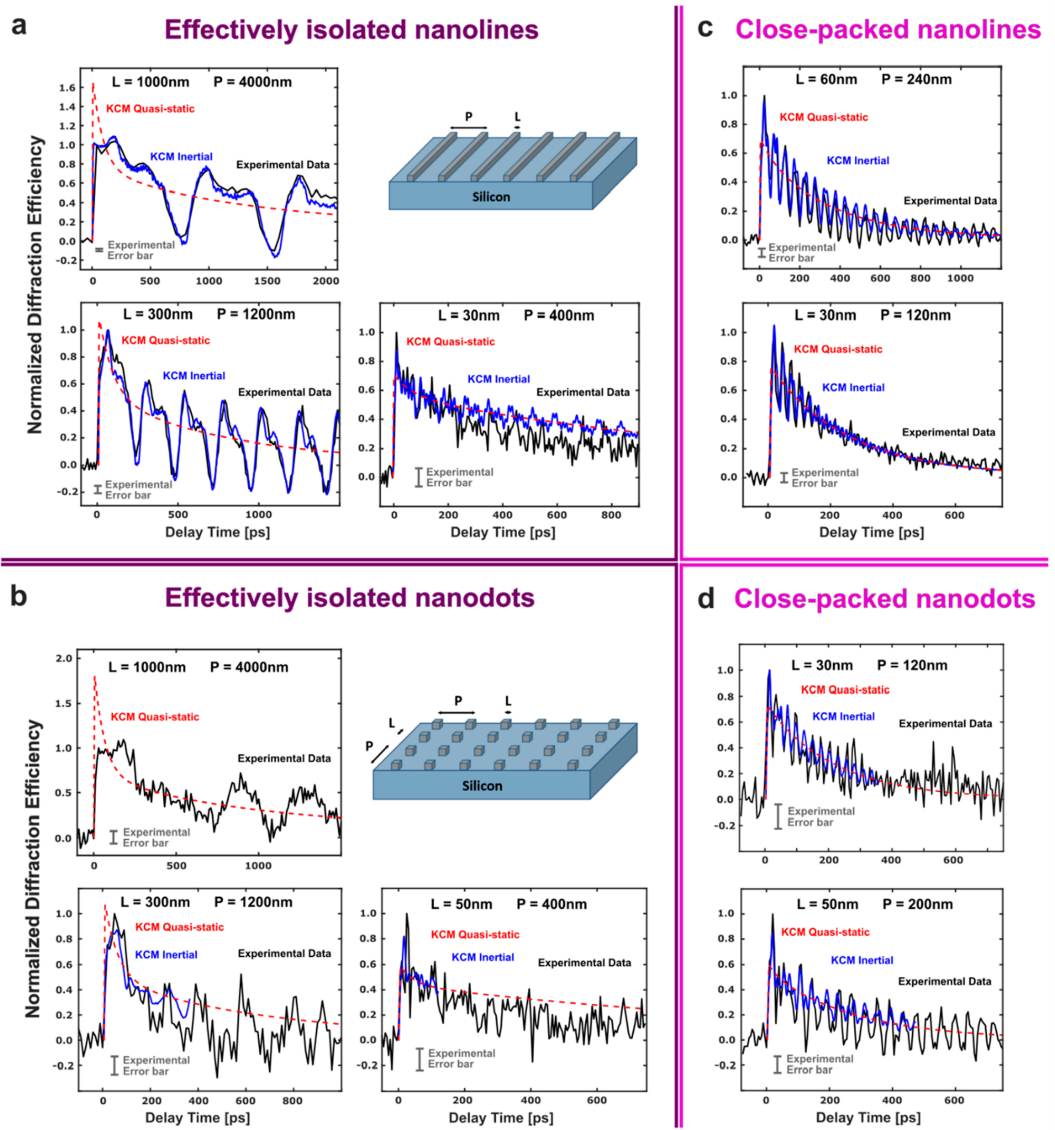
Direct comparison between EUV scatterometry data and KCM modeling in 1 and 2D. Experimental and theoretical normalized change in diffraction efficiency as a function of delay time for different sizes *L* and periods *P* for (a) effectively isolated, i.e., where (*P* – *L*) > 2*l*, nanolines (1D) and (b) nanodots (2D). Black lines denote experimental data where the error is represented by the gray bar. Blue lines indicate the inertial KCM predictions, and red lines denote the KCM quasi-static predictions which describe only the thermal transport without the contribution of oscillating elastic waves. Theoretical predictions are computed using the same geometry-independent parameters for all nanostructure sizes and shapes. The theoretical curves are identically normalized in each case so that the initial energy released to the heaters matches experiment (see Methods). Inertial simulations for nanodots are shown just in a short time window because of their high computational cost. We note that the first mechanical oscillation prevents observation of the initial temperature decay described by quasistatic curves (see Supplementary Section 1). Also shown are the experimental and theoretical changes in diffraction efficiency for close-packed, i.e., (*P* – *L*) < 2*l*, (c) nanolines (1D) and (d) nanodots (2D) of different sizes L and periods P. Theoretical results are solutions of eq 1 with *l*_eff_ = (*P* – *L*)/2, while the other parameters are the same size-independent values. The only fitting parameter for the entire data set is the intrinsic thermal boundary resistance, which is set to 2.25 nKm^2^/W for this work. The excellent agreement between KCM and the experimental data for the highly nondiffusive decay for both 1D- and 2D-confined heat source geometries demonstrates the predictive capability of this model.

## Results and Discussion

We first study effectively isolated heat sources for both 1D-confined (nanolines) and 2D-confined (nanodots) of different sizes and periodicities. Figure 2 compares the experimental results on nanolines and nanodots with theoretical KCM solutions obtained using COMSOL. We compare both inertial solutions, which include elastic waves generated by the impulsive pump laser excitation and quasi-static solutions without elastic waves to isolate the effects of the heat flow (see Supplementary Section 1). We use *ab initio* calculations to compute the intrinsic parameters of bulk silicon at *T* = 300 K: κ = 145 W/mK, τ = 50 ps, and *l* = 176 nm.^21,37^ For the thermal boundary resistance, which is an intrinsic property that depends only on the materials and the fabrication process, we use *R*_1_ = 2.25 nKm^2^/W for all nanostructure geometries (see Methods), which agrees with previous EUV scatterometry measurements on these samples^9^ and is close (∼2×) to the value obtained from time-domain thermoreflectance.^45^ We extracted this value from the large heater data, where size effects are negligible, and it is the only fitted parameter used for this data set. The excellent agreement in Figure 2 between experiment and theory demonstrates a significant advance in modeling; the nanoline thermal decay has already been shown to be highly nondiffusive^8,9^ and the models employing a suppression function are not easily calculable for a nanodot geometry.^5^ KCM—which is based on only a few key parameters—accurately predicts the thermal transport and elastic waves in both nanolines and nanodots without any geometry-dependent fit parameters, which is beyond the current capabilities of microscopic descriptions.

In the close-packed situation, (*P* – *L*) < 2*l*, nonlocal effects are expected to yield interaction between heaters, as phonons from a given source are able to reach neighboring sources before scattering. In this case, one does not expect eq 1 to be applicable since higher-order derivatives should be included in the transport equation.^35^ To keep the model as simple as possible, we propose that the effects of these higher-order terms can be absorbed into a geometry-defined value *l*_eff_, where eq 1 is still sufficient to describe the system. We propose the simplest expression that satisfies limiting cases: *l*_eff_ = (*P* – *L*)/2 (<*l*). For this expression, when the period *P* tends to the line width *L*, *l*_eff_ → 0. In this limit, the grating tends to a line of infinite line width, and thus, viscous effects should vanish. In the other limit, if (*P* – *L*) → 2*l*, we recover *l*_eff_ → *l* as constructed. Using this expression for *l*_eff_, we compare KCM predictions with experimental results for close-packed nanoline and nanodot heaters in Figure 2c,d. The model predicts that closely spaced heat sources cool faster than widely spaced ones, as uncovered in previous experiments.^6,8,9^ We also experimentally demonstrate that this same counterintuitive behavior observed in nanoline arrays is universal and manifests in nanodot arrays, since the *L* = 50 nm with *P* = 200 nm nanodot signal is relaxed at 800 ps while *L* = 50 nm with *P* = 400 nm is not. The excellent agreement between the KCM prediction and experimental results for the close-packed cases shows that KCM can model this behavior with a simple expression for *l*_eff_ (without fitting), while the other parameters used are the same used in the isolated cases. In summary, both nanoline and nanodot experiments can be predicted by the KCM using the intrinsic value *l* = 176 nm when sources are separated a distance larger than 2*l* (effectively isolated sources), and a geometry-defined effective value when distances are smaller (close-packed sources). This modification of *l* for a specific situation allows us to retain both the predictive capability and simplicity of the model.

**Figure 3:**
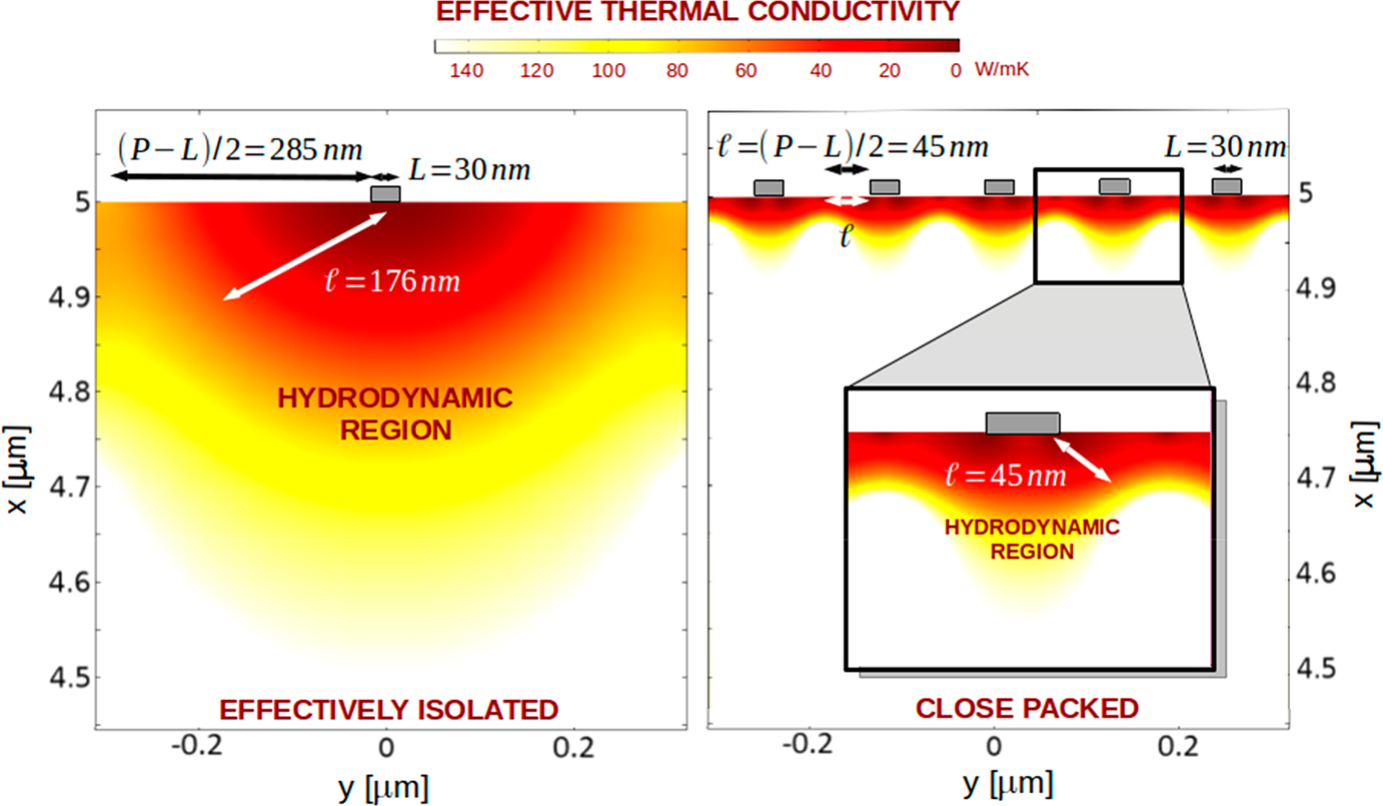
Hydrodynamic regions in effectively isolated and close-packed situations. Effective thermal conductivity profile on silicon, |*q*|/|∇*T*|, predicted by KCM for a nanoheater of width 30 nm at *t* = 0.5 ns for (left) isolated (*P* = 600 nm) and (right) close-packed (*P* = 120 nm) configurations. Similar to fluids, a friction-like reduction of thermal transport appears in the regions of the substrate where heat flux gradients are large. Parameter *l* defines the characteristic size of the region below heaters where these hydrodynamic effects are important (hydrodynamic region). When sources are separated a distance larger than 2*l* (effectively isolated lines), one uses the intrinsic value *l* = 176 nm. When this distance is smaller, i.e., (*P* – *L*) < 2*l*, an effective value *l*_eff_ = (*P* – *L*)/2 (<*l*) is used. The red color indicates regions where the thermal transport has been reduced (compared to diffusion) while the white color represents regions of diffusive transport. In close-packed configurations, the interaction between heaters homogenizes the profile, thus reducing viscous effects to a smaller region of size *l*_eff_. As a result, close-packed configurations evacuate heat faster than isolated lines of the same width as shown in ref (8). The profiles shown do not appreciably change during the time scale of experiments. Note that scales are the same in both panels.

Using our model, we interpret the behavior of the effectively isolated sources from a hydrodynamic viewpoint and compare it to the close-packed sources. For effectively isolated sources, hydrodynamic effects become relevant when line width *L* is on the same scale as the phonon mean free paths ∼*l*; thus, the non-Fourier terms in eq 1 reduce the heat flux, compared to Fourier’s law, in agreement with experiments.^5−9,12,44^ This phenomenon is analogous to a friction that arises from the large gradients in heat flux that impedes heat flow, referred to as a viscous resistance.^38^ In other words, when line width *L* is on the same scale as ∼*l*, there is not enough resistive phonon collisions to scatter the heat outward in all directions as diffusion assumes. Instead, the thermal energy is forced straight downward into the substrate over a distance related to ∼*l* before enough resistive phonon collisions occur to dissipate energy in all directions, shown schematically in Figure 1. These hydrodynamic-like friction effects resulting from a lack of resistive collisions have been described in other formalisms albeit with different interpretations. For example, models using a phonon suppression function predict heat flow that is less efficient than Fourier’s law when line width *L* is on the same scale as ∼*l*, similar to our hydrodynamic model; however, this phenomenon is interpreted as a reduced number of carriers due to ballistically traveling phonons.^14,34^ Additionally, models incorporating anisotropic behavior of thermal conductivity are parallel to the downward flux forcing predicted by our hydrodynamic model.^10^ The viscous term in eq 1 naturally includes both heat flux reduction and apparent anisotropy observed by experiments. In Figure 3, we visualize these substrate regions where viscous effects are important (hydrodynamic regions) by converting results to a spatially dependent effective thermal conductivity of silicon. Because of their proximity to the interface, if one tries to apply Fourier’s law, hydrodynamic effects might be interpreted either as an increase of the thermal boundary resistance^8^ or as a reduction of the thermal conductivity near the heater.^5,32^ In the effectively isolated case with *L* = 30 nm and *P* = 600 nm, this region has a size of order *l* ≃ 200 nm, while in the close-packed case (*P* = 120 nm), it is much smaller and of order *l*_eff_ ≃ 50 nm. Therefore, we hypothesize that the interaction of the nearby heat sources in the close-packed scenario reduces the nonlocal length, decreasing viscous effects, allowing the system to cool more efficiently than with isolated heaters. The microscopic description of this effect is the subject of future work.

**Figure 4:**
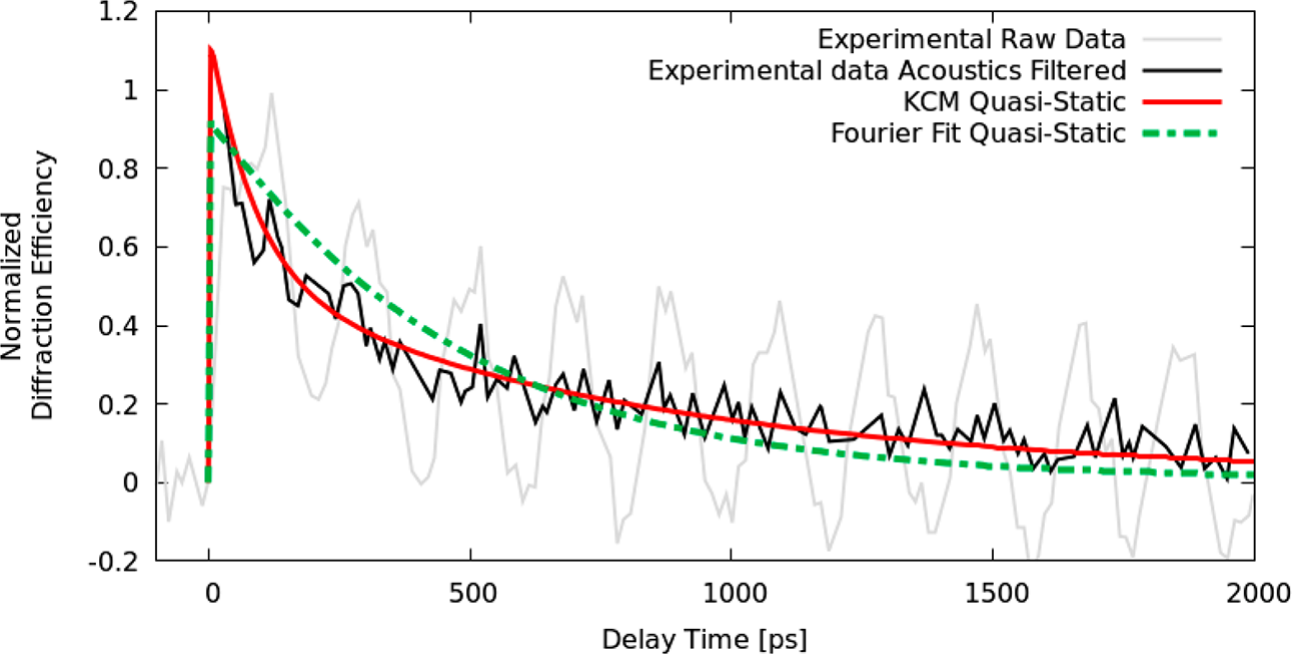
Experimental and theoretical quasi-static change in diffraction efficiency. Comparison of the thermal relaxation for effectively isolated heater lines of *L* = 250 nm and *P* = 1000 nm. The black (gray) line denotes experimental data without (with) acoustics, the red line is our KCM prediction using intrinsic parameters, while the green line is a Fourier model using an effective thermal boundary resistance value fitted to obtain the best match to data. The Fourier fit overestimates experimental decay at short times and underestimates it at long times. Experimental measurements indicate that the thermal decay of heaters cannot be described by just one characteristic time, like the prediction by Fourier’s model; however, KCM captures the decay for all times. Raw experimental data is from ref (8).

To demonstrate the advantages of our hydrodynamic model over the traditional effective Fourier model with a best-fit boundary resistance, we compare the two theoretical predictions to experimental data for the isolated 250 nm line width case in Figure 4. To emphasize the thermal decay of the system, we compare only the quasi-static calculations and data where the acoustic waves have been subtracted using the matrix pencil method (see Supplementary Section 3). Although an effective Fourier model can quantify the degree of the nondiffusive nature of the system, one finds that the best fit Fourier model fails to describe data at all times, as it overestimates the decay at the beginning and underestimates it at the end. In contrast, KCM predictions agree with data at all times. This plot indicates that the experimental results display two characteristic times: a fast one at short times and a slow one at longer times. These two different time scales are also apparent in the other nanostructure sizes shown in Figure 2. Therefore, as diffusive transport in these geometries contains only a single characteristic time scale, the effective Fourier model *cannot* capture the full nanostructure relaxation and misses the underlying physics, even with fitted intrinsic parameters (see Supplementary Section 2). We note that thermal transport data from visible probe techniques is typically not fitted until >100 ps after the pump pulse.^5,6^ Indeed, if the data from the first hundreds of picoseconds is excluded from our analysis, the presence of two distinct time scales cannot be observed (see Supplementary Section 2). In contrast, our EUV probe is only sensitive to surface deformations (see Methods) and thus precisely captures two distinct time scales—a signature of non-Fourier heat transport.

A distinct advantage of KCM is that we can gain deeper insight into the two time scales of thermal relaxation by investigating the role played by hydrodynamics. To do this, we analytically solve the thermal equations in the heater and the substrate for the case *L* < *l*. In this range, hydrodynamic effects are dominant: the *q* term in eq 1 can be neglected compared to the Laplacian term, and the heat flux obeys the (linear) Navier–Stokes equation. The system of equations obtained is
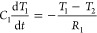
2
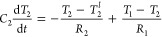
where *T*_1_ is the heater temperature, *T*_2_ the average temperature of the substrate at the interface, and *T*_2_^*l*^ the average substrate temperature in the outer part of the hydrodynamic region, i.e., at a depth of order *l* below the heater. *C*_1_ = *c*_h_*h* denotes the heat capacity of the heater per unit surface, with *c*_h_ and *h* the specific heat and height of the heater, respectively. *C*_2_ = *c*_s_*L*(1 + α)/*B* is a heat capacity per unit surface characterizing the substrate, with *c*_s_ the substrate specific heat, and *B* a calculated geometric coefficient that for nanolines is 3.0. *R*_1_ is the thermal boundary resistance between the metal and the substrate, and  is a size-dependent thermal resistance due to viscous effects (details in Supplementary Section 2). At short times, *T*_2_^*l*^ is close to *T*_2_^∞^ as heat has not reached this region, and eq 2 becomes a linear system with a double-exponential decay:
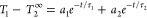
3with *τ*_*i*_ and *a*_*i*_ the characteristic times and weights, which are determined by *C*_1_, *C*_2_, *R*_1_, and *R*_2_. Therefore, KCM provides two characteristic times with specific expressions in terms of the physical properties of the system.

**Figure 5:**
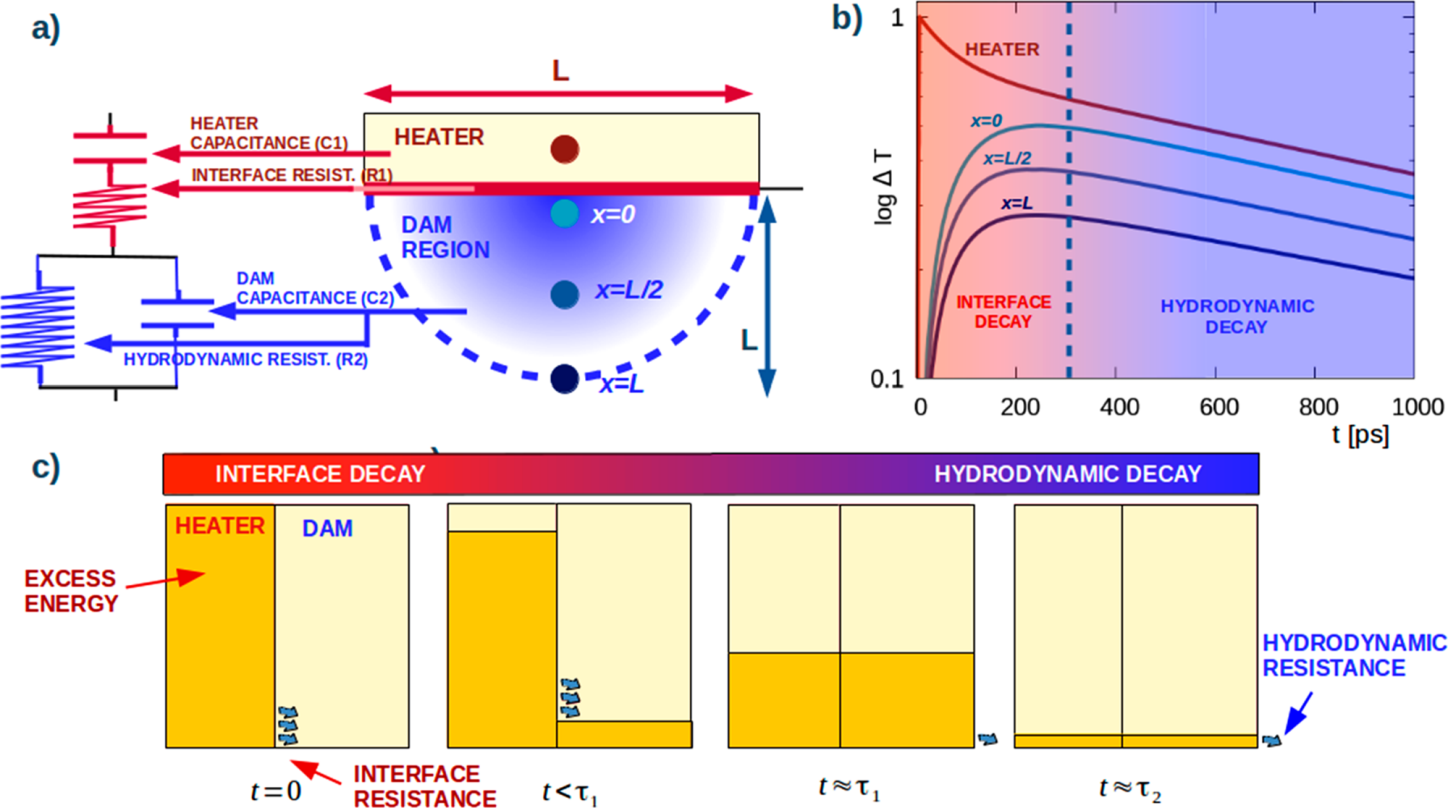
Two-box model for the thermal decay of heaters for *L* < *l*. (a) The energy released by the heater (with heat capacity per unit surface, *C*_1_) crosses the interface with the substrate at a rate determined by the thermal boundary resistance, *R*_1_. The thermal response of the substrate is determined by a region of size *L* below the heater—the dam region—which acts both as a heat reservoir of capacity *C*_2_, and as a thermal resistance *R*_2_ due to viscosity from hydrodynamic effects. An analogy to an equivalent electrical circuit is shown. (b) The temperature as a function of time is shown for the positions indicated in (a) from KCM solutions for *L* = 30 nm and *P* = 600 nm. At short times, the dam region retains the energy released by the heater and increases in temperature with a time scale τ_1_ dominated by the interface resistance. At larger times, a slow joint decay of heater and dam temperatures occurs with a characteristic time τ_2_ determined by the hydrodynamic resistance *R*_2_. (c) Cartoon of the two-box model in analogy with fluids. The two boxes represent the heater and dam with the water level indicating the temperature. For times less than τ_1_, the excess energy flows out of the heater into the dam through the interface resistance until temperatures equilibrate. For times on scale of τ_2_, excess energy in heater and dam escapes to the rest of the substrate at a rate ruled by hydrodynamic effects.

Equation 2 can be interpreted intuitively as a two-box model as seen in Figure 5. One box represents the heater, while the other box is a region of order *L* in the substrate below the heater (referred to as the dam region). The thermal response of the system begins when the heater is filled with thermal energy from the laser pulse. At short times after the laser pulse, the heater releases the energy into the dam region, which retains the energy and rapidly increases in temperature. The initial rate of this energy transfer is dominated by the intrinsic thermal boundary resistance between the heater and substrate. At larger times, when the dam region has equilibrated with the heater, the dissipation of the thermal energy is dominated by the rate of energy transfer out of the dam region into the rest of the substrate. Therefore, the substrate plays two roles in the thermal response of the system: it acts both as an energy reservoir with heat capacity *C*_2_ and as a thermal resistance *R*_2_. The rate of energy transfer in these later times is controlled by the viscous resistance, i.e., hydrodynamic effects. The thermal relaxation of the heaters can be described by an equivalent circuit (Figure 5a) and illustrated by a fluid analog (Figure 5c). The predicted temperature evolution of the system as a function of time and position are shown in Figure 5b and Supplementary Section 2.

For small isolated sources, we find simple expressions for the characteristic times, namely τ_1_ = *R*_1_*C*_eq_ = *R*_1_*C*_1_*C*_2_/(*C*_1_ + *C*_2_), and τ_2_ = (*C*_1_ + *C*_2_)*R*_2_. For nanolines of *L* = 50 nm, these expressions yield τ_1_ = 50 ps and τ_2_ = 1050 ps; thus, τ_2_ is an order of magnitude larger than τ_1_. In this limit, τ_1_ depends on the thermal boundary resistance, while the viscous time scale τ_2_ does not depend on the thermal boundary resistance but mainly on the nonlocal length *l* and geometry:
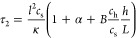
4

Therefore, for small isolated sources, KCM can provide simple analytical expressions for the two different time scales of the heat transfer, each one associated with a different resistive mechanism. This allows accurate experimental validation of the nonlocal length value for silicon at room temperature (a sensitivity analysis of various KCM parameters is provided in Supplementary Section 2). Additionally, the two-box model eq 2 can also be applied to close-packed experiments by substituting *l* by *l*_eff_; however, the simple expression of eq 4 cannot be used in this case (see Supplementary Section 2).

**Figure 6:**
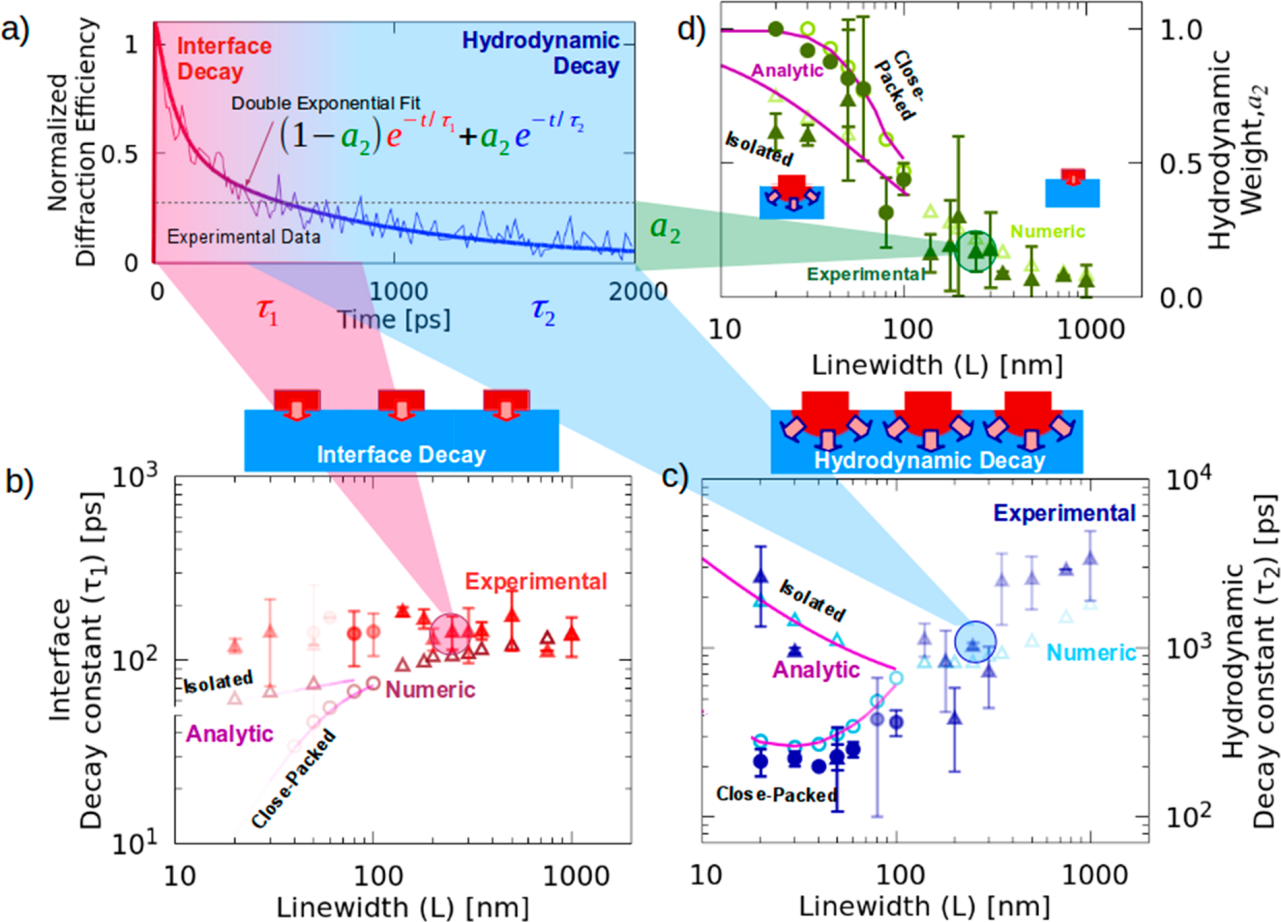
Two characteristic decay times in thermal relaxation of nanoline (1D) experiments. (a) The experimental change in diffraction efficiency, with oscillations removed, for a heater line of *L* = 250 nm and *P* = 1000 nm (thin line) can be fitted with a double exponential decay (thick line), from which two characteristic times are extracted: a short time scale (red line region, τ_1_) and a long time scale (blue line region, τ_2_). (b,c) Characteristic time τ_1_ and τ_2_ versus heater line widths L for effectively isolated (solid triangles) and close-packed (solid circles) experiments. KCM numerical (analytical) results are denoted by open symbols (lines). The color intensity in the symbols indicates the weight of each characteristic time in the overall decay. The short time scale (τ_1_ ∼ 0.1 ns) is dominated by the interface resistance, while the long one (τ_2_ ∼ 1 ns) is ruled by the hydrodynamic effects in the substrate. Additionally, the difference between the dissipation of close-packed versus effectively isolated heat sources is demonstrated. (d) The normalized weight of the hydrodynamic characteristic time in the temperature decay, *a*_2_ (= 1 – *a*_1_), is displayed versus line width for all experiments, showing the transition from interface- to hydrodynamic-dominated decay as source size decreases. These experimental fits include raw data from refs.^8,9^ and the current study.

Although the two-box model has been derived at small sizes, it also characterizes the non-Fourier behavior for all experimental sizes. To validate the intuition provided by the two-box model, we fit a double-exponential decay (eq 3) to each of our experimental measurements, as shown in Figure 6a. We compare the fits of experiments to fits of numerical KCM simulations and the analytical two-box model in Figure 6b–d. We find that the experimental fit results agree well with both KCM numerical and analytical calculations. Additionally, we confirm the existence of a short time scale (τ_1_ ∼ 100 ps) which is dominated by the intrinsic thermal boundary resistance in Figure 6b and a longer time scale (τ_2_ ∼ 1 ns) which is dominated by the hydrodynamic effects in Figure 6c. Figure 6c also displays the splitting of the decay times between effectively isolated and close-packed experiments, i.e., the increase in dissipation efficiency for close-packed heat sources. In Figure 6d, we plot the weight of the hydrodynamic dominated decay, *a*_2_ in eq 3, which shows a transition from a primarily hydrodynamic decay for small heaters, to a decay ruled by the thermal boundary resistance at large sizes. This is expected as large sizes should converge to the Fourier prediction, which contains a single time scale. Therefore, the size-*dependent* effective boundary resistance extracted by the effective Fourier model in refs (8 and 9) can be reinterpreted as capturing the weighted average of the time-scales (τ_1_, τ_2_) generated by a size-*independent* boundary resistance and size-*dependent* localized hydrodynamic effects.

## Conclusions

In conclusion, we have shown that by adding a hydrodynamic heat transport term, we can explain the thermal transport behavior of nanoscale metal–semiconductor samples with 1D- and 2D-confined heat source geometries over a large range of sizes, allowing a deeper insight of the physical behavior beyond effective Fourier’s law. The hydrodynamic model allows us to uncover two fundamental mechanisms in the thermal relaxation of nanoheaters on semiconductor and dielectric substrates: a first decay ruled by the interface followed by a second decay controlled by hydrodynamic heat transport in the substrate. For large nanoheater sizes, the former mechanism dominates, while for small nanoheater sizes, the hydrodynamic effects dominate. We have found compact analytical expressions for the time scales of these mechanisms and for the general thermal decay of heaters by developing a simple two-box model. In contrast to the single exponential decay predicted by Fourier for the semi-infinite substrate conditions of our experiments, the hydrodynamic model yields a two-exponential decay, which has been confirmed by extensive experiments in 1D- and 2D-confined source geometries. This two-exponential kinetics thus confirms the non-Fourier behavior of these experiments.

In contrast to previous models, the present hydrodynamic model contains no geometry-dependent fitting parameters, and it is thus predictive. The excellent agreement between KCM and the experimental data for the highly nondiffusive decay for both 1D- and 2D-confined heat source geometries demonstrates the versatility and generality of this model to capture behavior in complex, device-relevant geometries. In addition, our formalism enables predictive strategies to reduce the cooling time of nanoscale heaters. The mesoscopic character of the model and the use of intrinsic, geometry-independent parameters allows it to be easily extended to the complex architectures required by nanoscale technologies, where the lack of control on heat dissipation represents an important limitation for future developments.

## Methods

### EUV Dynamic Scatterometry Measurements

The sample consists of metallic Ni nanostructure arrays fabricated on the surface of a silicon substrate using an e-beam lithography technique. The nanostructure arrays are 150 × 150 μm^2^ areas consisting of both periodic nanolines and nanodots with line widths ranging from 1 μm down to 20 nm, periods ranging from 4 μm down to 80 nm, and average heights of 11.5 nm. The line width and period of the nanoline/nanodot arrays is independently controlled in order to separate the effects of size and spacing. The dimensions of the various arrays are characterized using atomic force microscopy (see Supplementary Section 4). To launch dynamics in the sample, an ultrafast infrared (780 nm wavelength, ∼25 fs pulse duration) pump beam is incident on the sample with ∼ 20 mJ/cm^2^ fluence and ∼ 275 μm spot size. The pump light is preferentially absorbed by the metallic nanostructures which causes rapid heating followed by impulsive thermal expansion in the nanostructures. The coherent excitation of the periodic arrays launches acoustics waves that propagate along the surface of the silicon substrate. As the heated nanostructures cool down by thermal dissipation into the substrate, they relax back to their original profile. An ultrafast, short wavelength probe beam is generated by focusing an ultrafast infrared pulse into an Ar filled glass capillary. A quantum nonlinear process called high harmonic generation converts a portion of the infrared light into a coherent short wavelength (∼30 nm) ultrashort pulse duration (∼10 fs) extreme ultraviolet (EUV) beam.^46^ The short wavelength of the probe allows for exquisite picometer sensitivity to the surface displacement and allows for measurements of 10s nm nanostructures.^47^ Moreover, these wavelengths interact with core electrons far from the Fermi surface, which are not affected by small temperatures changes as the photon energies are far from resonances in nickel.^47−49^ The probe beam is scattered from the nanostructure arrays at a set time delay, controlled by a mechanical delay stage, relative to the pump beam and captured on an EUV sensitive CCD camera. Images of the EUV scattering pattern with and without the pump beam are subtracted, allowing us to observe the change in the diffraction pattern. By subtracting the change in intensity of the reflected EUV light from the change in intensity of the diffracted EUV light, we can compute the change in diffraction efficiency. This change in diffraction efficiency is monitored as a function of time delay between the pump and probe beams and can be directly related to the surface deformation of the sample.

### Thermoelastic Modeling

The microscopic expressions required for the *ab initio* calculation of the KCM parameters can be found in refs (37 and 38). The same parameter values for silicon at room temperature have been used to model other experiments.^21,22,38,44^

The temperature and the heat flux are obtained by solving the energy conservation equation along with the heat transport equation (Fourier’s law for the heaters and eq 1 for the substrate). The second-order derivatives in the substrate transport eq (eq 1) require the inclusion of extra boundary conditions for the heat flux. A slip boundary condition relating the tangential heat flux in the substrate and its derivatives is imposed in the interfaces and in the silicon free surfaces (see Supplementary Section 1). In the free surfaces, thermal insulation is ensured by fixing normal component to zero. In the interfaces, we impose continuity of the heat flux normal component in the metal and in the substrate. Finally, we use a generalized boundary condition for the temperature jump in the interface including a Kapitza thermal boundary resistance term along with nonlocal terms.^22^ Using *ab initio* calculations, we compute a lower bound for the thermal boundary resistance assuming diffusive phonon reflections and perfect contact area (see Supplementary Section 1). However, the nanogratings fabrication process produces interface defects that increase the actual boundary resistance value. Therefore, a single correcting factor for the boundary parameters is required to predict the thermal decay of all the gratings (1D and 2D). The obtained correcting factor is fitted from the thermal decay of the largest experimentally available 1D grating (*L* = 1 μm) and hence does not depend on the model used. For large gratings, the hydrodynamic corrections do not play any role and we obtain the same boundary resistance correction using KCM or effective Fourier model. Specifically, we obtained a thermal boundary resistance value 3.1 times larger than the lower bound. This factor is similar to the one obtained in previous work for a similarly fabricated metal–semiconductor interface.^22^

The thermal equations are coupled with the classical elastic equations to predict the surface deformation of the system in order to compute the resulting change in diffraction efficiency using numerical Fresnel propagation. Specifically, the stress tensor of the nickel and the silicon includes a linear thermal expansion term. Moreover, the thermo-elastic energy exchange term is included in the energy conservation equation. For heaters, we use nominal bulk nickel elastic properties. For the substrate, we use an anisotropic stress tensor accounting for the structural defects generated during the fabrication of the nanogratings on the substrate top surface.^50^

All the parameter values used and a detailed explanation of the thermoelastic equations and the boundary conditions can be found in Supplementary Section 1.

### Comparison between Experiment and Model Predictions

Since KCM consists of a linear set of partial differential equations, the surface deformation and the predicted diffraction efficiency linearly depends on the amount of energy deposited in the heater by the laser pulse. In the simulations, a uniform energy density of 1W/m^3^ with a duration of <2.5 ps is introduced in the heater. To compare the model predictions and the experiments, the diffraction efficiency obtained in KCM inertial simulation is scaled by a factor to match the first experimental peak. This is equivalent to scaling the simulated energy density by this factor and this same scaling is used to normalize the quasi-static simulations. This procedure is also applied to the effective Fourier simulations in order to compare Fourier and experiments in Figure 4. Note that a slight correction factor has been added to scaling of the quasi-static simulation of *L* = 30 nm with *P* = 120 nm in Figure 2d due to a small numerical error in the first few picoseconds of this inertial simulation.

The quasi-static solutions are obtained by removing inertial elastic effects, i.e., dynamic equilibrium is imposed during all the simulation (see Supplementary Section 1). These solutions capture the deformations just due to thermal expansion and hence can be used to track the temperature evolution of the system (see ref (9)). Note that the initial peak obtained in the quasi-static simulations is not observed in experiments because the system needs a finite time to expand.

### Double Exponential Fitting to Experimental and Numerical Data

For Figure 6, we performed double-exponential fits to both the experimental data and the numerical simulations. The quasi-static numerical solutions can be easily fit to a double-exponential using nonlinear least-squares; however, due to the noise and inertial elastic effects, a double-exponential function with four free parameters is too unconstrained to reliably fit to the experimental data. Therefore, we constrain the number of free parameters in a single fit while still independently extracting the four parameters of the double-exponential. We achieve this by fitting the data in several different steps. We determine a cut time, *t*_c_, to divide the experimental trace into two parts in time to separately fit the two exponentials. We define the cut time as the time when the ratio between the two exponentials is 1% and compute *t*_c_ using the fit values from the numerical solution. We fit the experimental data for times *t* > *t*_c_ to extract the longer decay time exponential; however, the functional form of the decay for large *t* is not purely single exponential. Because diffusive transport occurs far from the heat source at large *t*, the decay has a power law component superimposed on the exponential. To mitigate the effects of this power law on the extraction of the decay constant, we fit an effective Fourier model—with only two free parameters of effective thermal boundary resistance (*R*_eff_) and the overall normalization (*A*_2_^fit^)—for *t* > *t*_c_ and truncate the fit at roughly 2 times the expected decay constant. We can then convert *R*_eff_ to a decay time, τ_2_^fit^, since τ_2_^fit^ = *R*_eff_*c*_h_*h*.

To extract the other exponential, we correctly set the overall normalization by accounting for the inertial elastic effects in the experimental data. To do this, we fit the KCM quasi-static simulation to the experimental data with the acoustics waves subtracted for times *t* < *t*_c_, constraining the maxima of the inertial KCM simulation and experimental data to be within the experimental noise. The resulting maximum of the KCM quasi-static simulation, *A*, allows us to compute *A*_1_^fit^ = *A* – *A*_2_^fit^. We can extract the final parameter by fitting a double-exponential, *A*_1_^fit^*e*^–*t*/τ_1_^fit^^ + *A*_2_^fit^*e*^–*t*/τ_2_^fit^^, for *t* < *t*_c_ with only one free parameter, τ_1_^fit^. For Figure6b-d, we plot τ_1_ = τ_1_^fit^, τ_2_ = τ_2_^fit^, and *a*_2_ = *A*_2_^fit^/*A*, respectively, for both the experimental data and the numerical solutions. The error bars on the experimental data are the standard deviation from multiple measurements (if there are no error bars, then only one measurement was included). As the amplitudes of the exponentials (*a*_1_, *a*_2_) decrease, the extracted corresponding decay times (τ_1_, τ_2_) becomes more inaccurate. This is partially responsible for the difference between numerical solutions and experimental data for τ_2_ at large *L* and τ_1_ at small *L*. Additionally, inertial effects and noise affect the extracted values of τ_1_ as *L* decreases (see Supplementary Section 5).

## Supporting Information Available

Full details of the thermoelastic model (system of partial differential equations, boundary conditions, and parameter values), analysis of the heater thermal decay and derivation of the two-box model, details about Matrix Pencil Method and Atomic Force Microscopy, and discussion on the experimental extraction of the heater thermal decay times (PDF)
A preprint version of the present manuscript is available.^51^
