# Supplementary material for: A General and Predictive Understanding of Thermal Transport from 1D- and 2D-Confined Nanostructures: Theory and Experiment
Source: ACS Nano. 2021 Jul 30;15(8):13019–30. doi: 10.1021/acsnano.1c01946 (PMC8483436; doi:10.1021/acsnano.1c01946)
Supplement: Supplementary file 1 — nn1c01946_si_001.pdf [file nn1c01946_si_001.pdf]

SUPPLEMENTARY INFORMATION

A General and Predictive Understanding of Thermal Transport from 1D- and 2D-Confined Nanostructures: Theory and Experiment.

Albert Beardo*,†, Joshua L. Knobloch*,†, Lluc Sendra, Javier Bafaluy, Travis D. Frazer, Weilun Chao, Jorge N. Hernandez-Charpak, Henry C. Kapteyn, Begoña Abad, Margaret M. Murnane, F. Xavier Alvarez, Juan Camacho*

Contents

1 Thermoelastic Model **1**

2 Thermal Decay Analysis **5**

3 Matrix Pencil Method **17**

4 Atomic Force Microscopy **18**

5 More Details on Extraction of Decay Times **19**

1 Thermoelastic Model

The model equations can be solved using Finite Elements methods to determine the evolution of the displacement vector $\vec{u}$, the temperature T and the heat flux $\vec{q}$ in the metalic domains and the substrate, which allow comparision with the experimental measurements. The set of equations is the following:

Linear Elastic Equations

To describe the mechanic evolution of the system we use the elastic equation including inertial effects [1]

$$\rho \frac{\partial^2 \vec{u}}{\partial t^2} = \nabla \cdot \sigma, \tag{1}$$

where ρ is the density and σ is the stress tensor of the material.

For the heaters we use the bulk nickel stress tensor (isotropic) with linear thermal expansion:

$$\sigma_{ik} = K_{\text{Ni}}[-\alpha_{\text{Ni}}(T - T_0) + \nabla \cdot \vec{u}]\delta_{ik} + \mu_{\text{Ni}}[\frac{\partial u_i}{\partial x_k} + \frac{\partial u_k}{\partial x_i} - \frac{2}{3}\nabla \cdot \vec{u}\, \delta_{ik}] \tag{2}$$

where α_{Ni}, K_{Ni} and μ_{Ni} are the nickel coefficient of thermal expansion, the compressibility modulus and the shear modulus, respectively, and $T_0 = 300\text{K}$ is the ambient temperature.

For the silicon stress tensor, we also assume linear thermal expansion and we use an anisotropic form to include the effects coming from the stress generated by the nanolines on the substrate top surface [2]. The characterization of this stress tensor has been reported elsewhere [3] and it is necessary to reproduce the frequency of the mechanic oscillations:

$$\sigma = D : [\nabla\vec{u} - \alpha_{\text{Si}}(T - T_0)\mathbb{I}] \tag{3}$$

where α_{Si} is the silicon coefficient of thermal expansion, $\mathbb{I}$ is the identity matrix and D is the anisotropic elasticity matrix.

In order to obtain the quasi-static relaxation (*i.e.* suppress the acoustic oscillations) we substitute equation (1) by $0 = \nabla \cdot \sigma$.

Heat Transport Equations

Heat transport is modeled including the corrections of the phonon hydrodynamic model in the region where heat is carried mainly by phonons *i.e.* the silicon substrate.

Nickel Nanostructures (Fourier). Heat transport in metal lines or dots (height $h = 11.5\text{nm}$ and width L) is carried by electrons. As their mean free paths are much shorter than the sizes of the nanostructures, nonlocal effects are not expected. Consequently, Fourier's law is valid on these domains, in which we denote the heat flux and the temperature with subindex 1.

We include the linear thermoelastic coupling in the energy conservation equation to model the transfer between thermal and elastic energy:

$$c_{\text{Ni}}\frac{dT_1}{dt} + \nabla \cdot \vec{q}_1 = Q - p_{\text{Ni}}\frac{\partial}{\partial t}\nabla \cdot \vec{u} \tag{4}$$

where c_{Ni} is the specific heat of Nickel and $p_{\text{Ni}} = \alpha_{\text{Ni}}K_{\text{Ni}}T_0$. The heating energy density Q is $1W/m^3$ for time $t < 2.5ps$ and 0 otherwise. The normalized thermal response of the system does not depend on the used value for Q due to the linearity of the model equations. The temporal duration of the pulse is estimated using a two-temperature model [4, 5]. Moreover, we confirmed that the exact length of the heat pulse (0-10ps duration) does not significantly affect the results.

For the transport equation we use the Fourier's law

$$\vec{q}_1 + \kappa_{\mathrm{Ni}} \nabla T_1 = 0 \tag{5}$$

where κ_{Ni} is the bulk thermal conductivity of Nickel.

Silicon substrate (Hydrodynamic Transport). We use the energy conservation equation with linear thermoelastic coupling and without the source term,

$$c_{\mathrm{Si}} \frac{dT}{dt} + \nabla \cdot \vec{q} = -p_{\mathrm{Si}} \frac{\partial}{\partial t} \nabla \cdot \vec{u} \tag{6}$$

where c_{Si} is the specific heat of silicon and $p_{\mathrm{Si}} = \alpha_{\mathrm{Si}} K_{\mathrm{Si}} T_0$.

For the heat transport we use the GKE including non-local and memory effects,

$$\tau \frac{\partial \vec{q}}{\partial t} + \vec{q} + \kappa_{\mathrm{Si}} \nabla T = \ell^2 (\nabla^2 \vec{q} + \alpha \nabla \nabla \cdot \vec{q}) \tag{7}$$

where κ_{Si} is the silicon bulk thermal conductivity, ℓ is the non local length, τ is the heat flux relaxation time and α is a dimensionless parameter. Equation (7) can be derived from the BTE assuming an averaged phonon mode relaxation time with $\alpha = 1/3$ [6] and for the collective regime (normal dominant phonon collisions) with $\alpha = 2$ [7]. In consistency with [8, 9, 10, 11], here we propose the use of an effective form of equation (7) by considering interpolated parameters κ, ℓ, τ between the kinetic (resistive dominant phonon collisions) and the collective situations at the reference temperature T_0, and $\alpha = 1/3$. Details of the interpolation and the *ab initio* calculation of these parameters can be found elsewhere [12, 9].

Boundary Conditions

Nanostructure Free surfaces Thermal insulation is imposed by setting to zero the normal heat flux component

$$\vec{q}_1 \cdot \vec{n} = 0 \tag{8}$$

where $\vec{n}$ is the boundary normal vector.

Interface On one hand we impose continuity of the normal component of the heat flux going through the interface:

$$\vec{q}_1 \cdot \vec{n} = \vec{q} \cdot \vec{n} \tag{9}$$

where $\vec{n}$ is the interface normal vector pointing towards the semiconductor.

On the other hand, we impose the temperature jump boundary condition that accounts for the nonequilibrium effects introduced by the interface (assuming diffusive reflections):

$$(T - T_1) = -R_1 \vec{q} \cdot \vec{n} + \frac{1}{\gamma^{\mathrm{Si}}} (\beta \nabla \cdot \vec{q} - \nabla \vec{q} : \chi) \tag{10}$$

where β, χ are *ab initio* calculated characteristic lengths. Derivation of this boundary condition and the explicit microscopic expression for these parameters by imposing energy balance restrictions with the use of the specific non-equilibrium distribution function of each domain can be found elsewhere [11]. The tensor χ is diagonal. A lower bound for the thermal resistance value R_1 is also obtained in [11] by assuming phonon diffusive boundary scattering and perfect interface (no reduction of the contact area between nickel and silicon due to fabrication defects),

$$R_1^{\mathrm{min}} = \frac{1}{2}(\frac{1}{\gamma_0^{\mathrm{Si}}} + \frac{1}{\gamma_0^{\mathrm{Ni}}}). \tag{11}$$

where $\gamma_0^{\mathrm{Si}} = \frac{1}{4}c_{\mathrm{Si}}v_{\mathrm{Si}}$ and $\gamma_0^{\mathrm{Ni}} = \frac{1}{4}c_{\mathrm{Ni}}v_{\mathrm{Ni}}$ (with v the average phonon group velocity for each material respectively). The actual thermal resistance value R_1 is unknown due to the lack of knowledge of the interface defects. Therefore we fit a correction factor $R_1/R_1^{\mathrm{min}} = 3.11$ to the data obtained from the largest experimentally available gratings $L > 750$nm, in which hydrodynamic effects are not relevant (*i.e.* Fourier and KCM predictions coincide). Hence, this correction does not depend on the model used. The second and third terms in the right-hand side of expression (10) are required to properly describe the non-local effects induced by the interface in the substrate heat flux and temperature profiles. In the present work only cause minor corrections for the smallest linewidths $L < 50$nm. The same correction factor has been applied to the resistance weighting the non-local term $\gamma_0^{\mathrm{Si}}/\gamma^{\mathrm{Si}} = 3.11$.

Slip boundary conditions For silicon, where a second order derivative of the heat flux is included on the thermal transport equation, a boundary condition for the tangential flux $\vec{q}_t$ is required. We use the slip boundary condition both in the silicon interface and free surfaces [13, 10]

$$\vec{q}_t = C\ell \, \nabla\vec{q}_t \cdot \vec{n} \tag{12}$$

where

$$C = \frac{1+p}{1-p}, \tag{13}$$

p is the fraction of specularly reflected phonons in the boundaries (details can be found in [10]). In consistency with (10) here we consider diffusive reflections $p = 0$, *i.e.* $C = 1$. In the silicon free surface, insulation is also imposed

$$\vec{q} \cdot \vec{n} = 0 \tag{14}$$

Substrate Only a geometry periodically repeated unit is simulated by imposing periodic boundary conditions. The reference temperature $T_0 = 300$K and null displacement vector $\vec{u} = 0$ is fixed in the substrate base.

Table 1: Thermoelastic model parameter values at 300K.

	Nickel	Silicon
ρ [$\mathrm{Kg\,m^{-3}}$]	8900	2329
α[$\mathrm{K^{-1}}$]	$12.77{\cdot}10^{-6}$	$3{\cdot}10^{-6}$
K [Pa]	$175{\cdot}10^{9}$	$95{\cdot}10^{9}$
μ [Pa]	$76{\cdot}10^{9}$	$52{\cdot}10^{9}$
c [$\mathrm{J\,m^{-3}\,K^{-1}}$]	$4{\cdot}10^{6}$	$1.6{\cdot}10^{6}$
κ [$\mathrm{W\,m^{-1}\,K^{-1}}$]	91	145
τ [s]	-	$50{\cdot}10^{-12}$
ℓ [m]	-	$176{\cdot}10^{-9}$

Parameter Values

Regarding the interface boundary condition, the calculated conductance bounds are $\gamma_0^{\mathrm{Si}} = 1068\mathrm{MW/m^2K}$ and $\gamma_0^{\mathrm{Ni}} = 1960\mathrm{MW/m^2K}$, and the thermal boundary resistance used is $R_1 = 2.25\mathrm{nK\,m^2/W}$. The tensor χ is diagonal with $\chi_{xx} = -31\mathrm{nm}$, $\chi_{yy} = \chi_{zz} = -16\mathrm{nm}$ where x denote the normal direction pointing towards the semiconductor. The length $\beta = -21\mathrm{nm}$.

Regarding the silicon stress tensor, we use an anisotropic model in Voigt notation $\{yy, xx, zz, xz, yz, zx\}$ with the following rotated tensor $D_{11} = 203, D_{12} = 66.5, D_{22} = 173, D_{13} = 36.5, D_{23} = 66.5, D_{33} = 203, D_{14} = 0, D_{24} = 0, D_{34} = 0, D_{44} = 83, D_{15} = 0, D_{25} = 0, D_{35} = 0, D_{45} = 0, D_{55} = 53, D_{16} = 0, D_{26} = 0, D_{36} = 0, D_{46} = 0, D_{56} = 0, D_{66} = 83$ [GPa] to match the simulated geometry that y is along [001] and x (interface normal direction) is along [110] in the silicon crystal.

The rest of parameter values used in the model for each material can be found in the Table 1.

2 Thermal Decay Analysis

In this section, we compare the non-equilibrium evolution of the system according to the Fourier model and to the Kinetic Collective Model (KCM) and we derive the two-box model equations from the KCM analytical solutions in the Stokes regime ($L < \ell$).

Fourier Model

First consider diffusive heat transport both in the Si substrate and in the Ni heater along with a Kapitza interface boundary condition with resistance R. We use the bulk thermal properties from Table 1 and we denote the thermal diffusivity of Nickel $\chi_{\mathrm{Ni}} = \kappa_{\mathrm{Ni}}/c_{\mathrm{Ni}} = 2.2{\cdot}10^{-5}\mathrm{m^2\,s^{-1}}$ and of silicon $\chi_{\mathrm{Si}} = \kappa_{\mathrm{Si}}/c_{\mathrm{Si}} = 9{\cdot}10^{-5}\mathrm{m^2\,s^{-1}}$. For illustration purposes, we discuss this benchmark model considering the spe-

cific case of heater height $h = 10\text{nm}$ and width $L = 20\text{nm}$, with $R = 1\text{nK m}^2/\text{W}$. In Figure 1 we show the corresponding temperature evolution of the heater obtained with COMSOL Multiphysics.

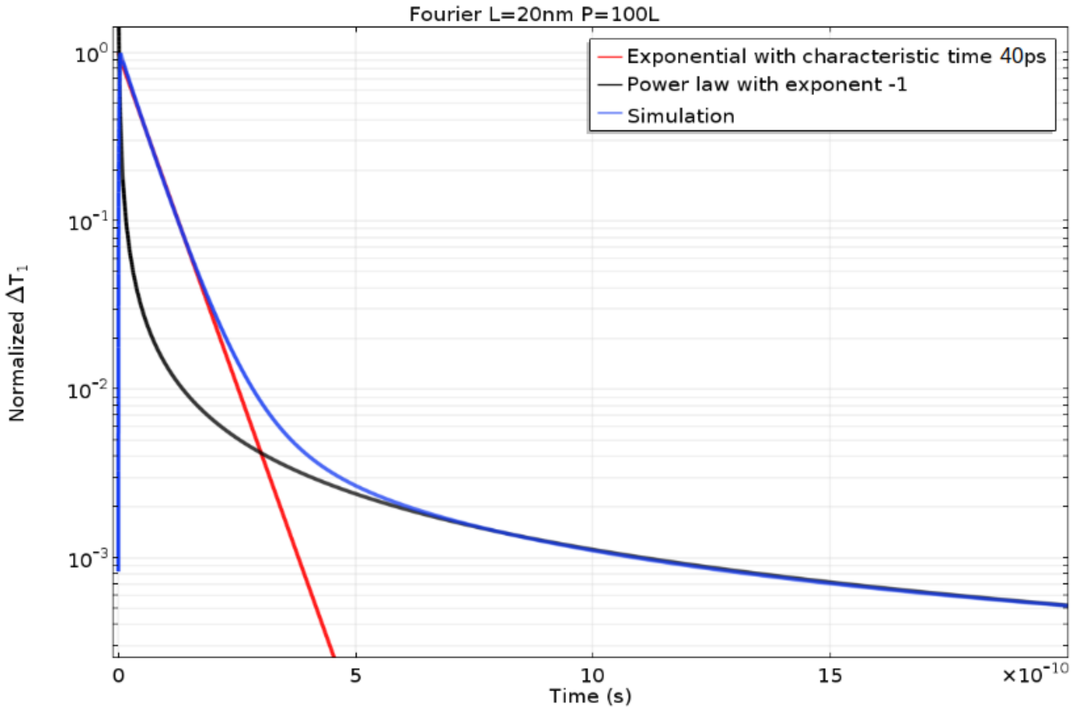

Figure 1: Heater temperature evolution for $h = 10\text{nm}$, $R = 1\text{nK m}^2/\text{W}$, $L = 20\text{nm}$ and $P = 100L$ according to Fourier model. The initial decay is fitted using an exponential and the long-time decay is fitted using a power-law.

The time scale of the thermal evolution in the heater is extremely fast $h^2/\chi_{\text{Ni}} = 4.5\text{ps}$ and hence the temperature in the heater is almost uniform within the time scale of the experiment. In the substrate, at time t, diffusion has penetrated a region of size $\sqrt{t\chi_{\text{Si}}}$. We can quantify an effective thermal resistance due to diffusion $r(t) = \sqrt{t\chi_{\text{Si}}}/\kappa_{\text{Si}}$. At early times $R > r(t)$, so the thermal decay is dominated by the interface. At times larger than $R^2\kappa_{\text{Si}}c_{\text{Si}} = 232\text{ps}$, we have $r(t) > R$ thus the thermal decay is dominated by substrate diffusion.

For $t < R^2\kappa_{\text{Si}}c_{\text{Si}}$: The heat flux in the interface is $|\vec{q}| = \Delta T/R$, where ΔT is the temperature difference between the heater and the substrate across the interface. Moreover, the heat flux leaving the heater can be estimated as $|\vec{q}| \sim -c_{Ni}h\frac{dT}{dt}$, and hence $\Delta T \sim -c_{Ni}hR\frac{dT}{dt}$. Therefore, the temperature evolution of the heater is an exponential with characteristic time $\tau_F = c_{Ni}hR = 40\text{ps}$.

For $t > R^2\kappa_{\text{Si}}c_{\text{Si}}$: Substrate diffusion has no characteristic time scale (infinite substrate) and hence the thermal decay follows a power law with an exponent depending on the space dimensionality. The thermal evolution in the region below the heater is instantaneous $L^2/\chi_{\text{Si}} = 4.4\text{ps}$ and hence we can consider that the heated region is a point and the temperature evolution is 2D (exponent -1).

In summary, the Fourier model predicts an initial exponential thermal decay

with characteristic time $\tau_F = c_{Ni} hR = 40$ps followed by a power law decay with exponent -1. The transition between both decays is estimated to be at time $R^2 \kappa_{\rm Si} c_{\rm Si} = 232$ps.

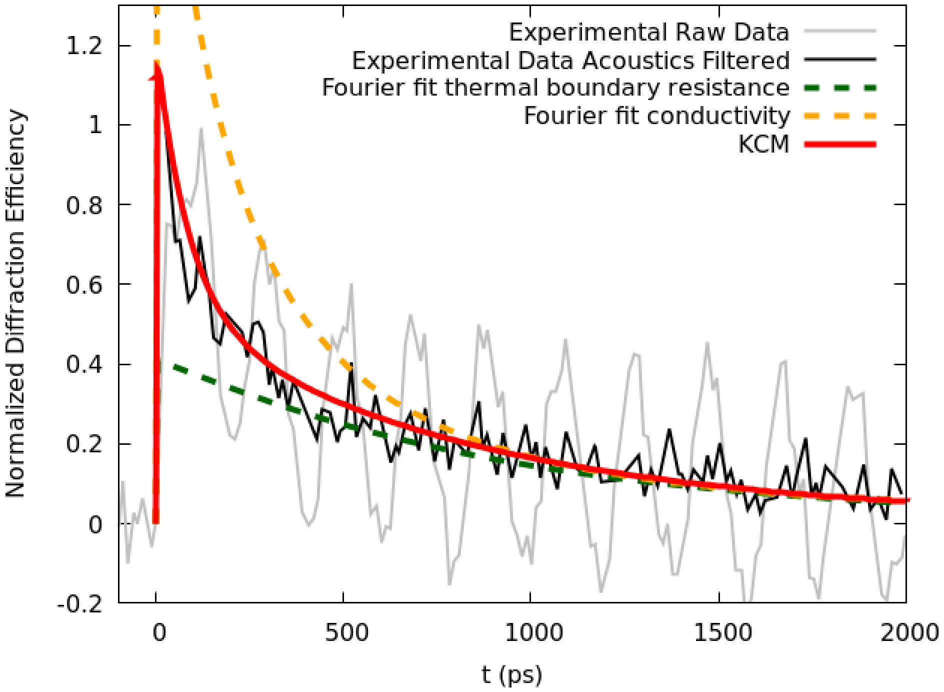

Figure 2: Fourier fits to experimental data restricted to $t > 500ps$ for a heater line of 250nm. The TBR fit ($R = 19\mathrm{nK\,m^2/W}$) using the intrinsic value for the substrate conductivity underpredicts the temperature for $t < 500ps$, whereas the conductivity fit ($\kappa = 0.4\kappa_{bulk}$) using the intrinsic value for the TBR overpredicts it. In both models, the predicted fraction of energy evacuated from the heater is substantially distorted.

As shown in Figure 4 of the main text, the functional form of the thermal decay according to Fourier's law is not consistent with the experimental decay from EUV scatterometry measurements, where a double exponential decay with two distinct characteristic times is observed (see also Figure 6 of the main text). Care must be taken when comparing our results with those from time-domain thermal reflectance (TDTR) [14], which are two completely different techniques. One key difference is that visible-based probe experiments often need to limit the analysis to the region $t > 500$ps due to the challenges separating the contributions of out-of-equilibrium electrons and thermal decay. However, our EUV probe does not suffer from this limitation as we measure the surface deformation through diffraction, which is not altered by the presence of nonequilibrium electrons. If we take EUV scatterometry data and exclude from our analysis the measurements for $t < 500$ps, one of the decay times ($\sim$100ps) reported in this work cannot be observed. Consequently, a diffusive model could fit the experimental data by excluding the initial timescales, as done in previous works like [14]. In Figure 2, we show two different fits using Fourier with an effective TBR and an effective substrate conductivity, respectively, restricted to $t > 500$ps. Both

approaches can reproduce the tail of the decay but fail to reproduce the initial system response. After the first nanosecond, these models are able to reproduce just the last 20% of the signal amplitude but cannot predict the other 80%, *i.e.* the largest part of the energy dissipation from heaters which is also the most important for applications.

Kinetic Collective Model

We assume now diffusive heat transport in the heater and hydrodynamic heat transport in the substrate using the Kinetic Collective Model as explained in Supplementary Section 1. In figure 3, we show an example of the temperature evolution of the heater for $L = 30$nm and $P = 400$nm obtanied with COMSOL.

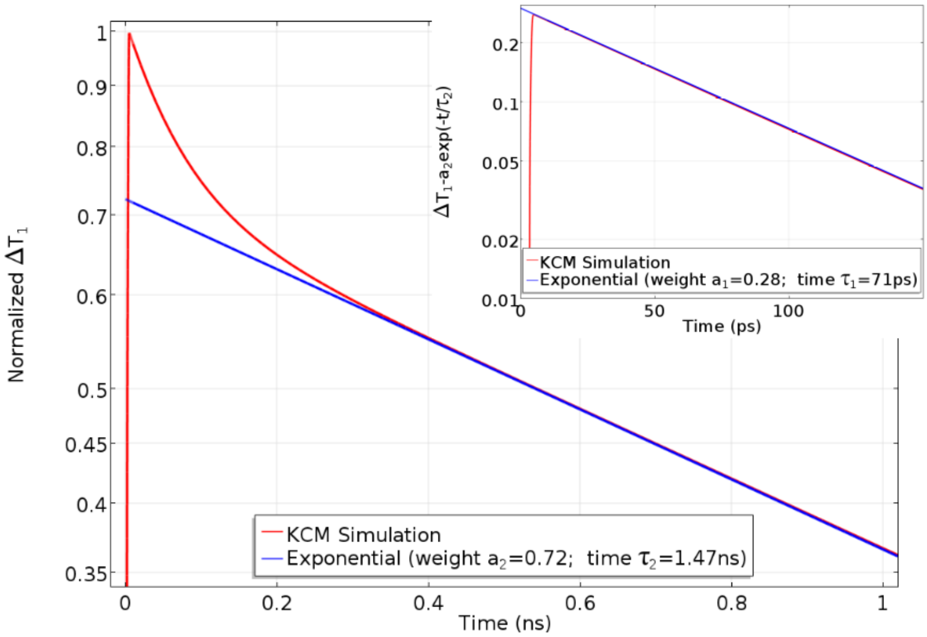

Figure 3: Heater temperature evolution for $L = 30$nm and $P = 400$nm according to KCM. The thermal decay is fitted with a double exponential.

In contrast to the Fourier model, the KCM predicts a slower thermal decay that can be fitted using a double exponential within the time scale of the experiment. We denote as τ_1, τ_2, a_1, a_2 the characteristic times and weights of the first and the second exponentials, respectively. The geometrical dependencies of these coefficients are represented in Figure 6 of the main text.

Description of Figure 6 and sensitivity of the KCM parameters

The information condensed in Figure 6 of the main article (reproduced here as Figure 4) is more easily understandable by identifying all the double exponential

decay coefficients for an specific sample and analyzing the sensitivity of the decay to changes in the KCM parameters values. Here we consider two examples that represent two different situations. The selected cases are represented in blue and red in Figure 4.

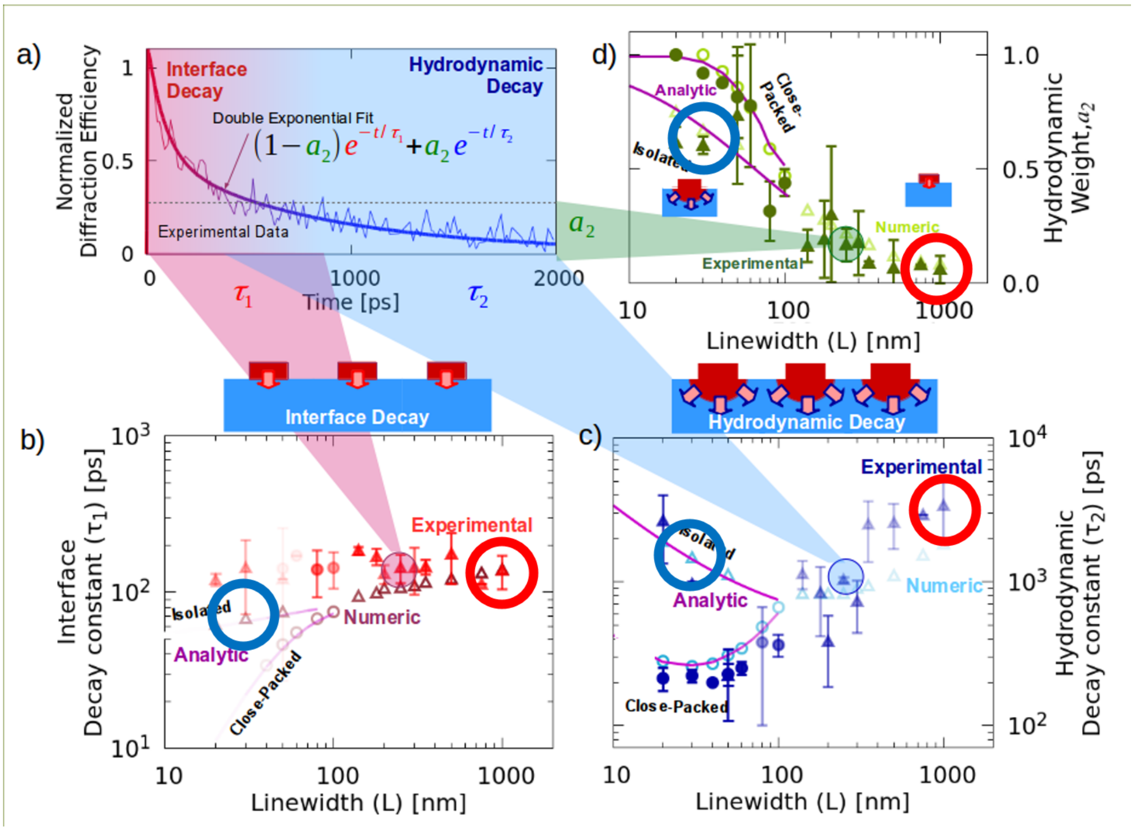

Figure 4: Two characteristic decay times in thermal relaxation of nanoline (1D) experiments. (a) The experimental change in diffraction efficiency, with oscillations removed, for a heater line of $L = 250$nm and $P = 1000$nm (thin line) can be fitted with a double exponential decay (thick line), from which two characteristic times are extracted: a short time scale (red line region, τ_1) and a long time scale (blue line region, τ_2). (b,c) Characteristic time τ_1 and τ_2 *versus* heater linewidths L for effectively isolated (triangles) and close-packed (circles) experiments. KCM numerical (analytical) results are denoted by open symbols (lines). The color intensity in the symbols indicates the weight of each characteristic time in the overall decay. The short time scale (τ_1 ~0.1ns) is dominated by the interface resistance, while the long one (τ_2 ~1ns) is ruled by the hydrodynamic effects in the substrate. Additionally, the difference between the dissipation of close-packed *versus* effectively isolated heat sources is demonstrated. (d) The normalized weight of the hydrodynamic characteristic time in the temperature decay, a_2(=1$-a_1$), is displayed *versus* linewidth for all experiments, showing the transition from interface- to hydrodynamic-dominated decay as source size decreases.

The blue circles indicate the coefficients of the double exponential decay for isolated small heaters with size $L = 30$nm and periodicity $P = 400$nm ($\tau_1 = 68$ps, $\tau_2 = 1470$ps, $a_2 = 1 - a_1$ =0.7). The thermal evolution for this sample is repre-

sented with blue lines in the two top plots of Figure 5. The influence of modifying the non-local length ℓ or the boundary resistance value R_1 is displayed in the left and right plots, respectively. Note that the smaller time τ_1 is determined by R_1 and the larger one τ_2 by ℓ, so each decay is associated to a different mechanism. In this case the weight $a_2 > 0.5$ and the hydrodynamic time is larger than the TBR dominated time ($\tau_2 > \tau_1$). Therefore, the heater thermal evolution is more sensitive to the ℓ value than to the interfacial resistance value R_1 as shown in Figure 5.

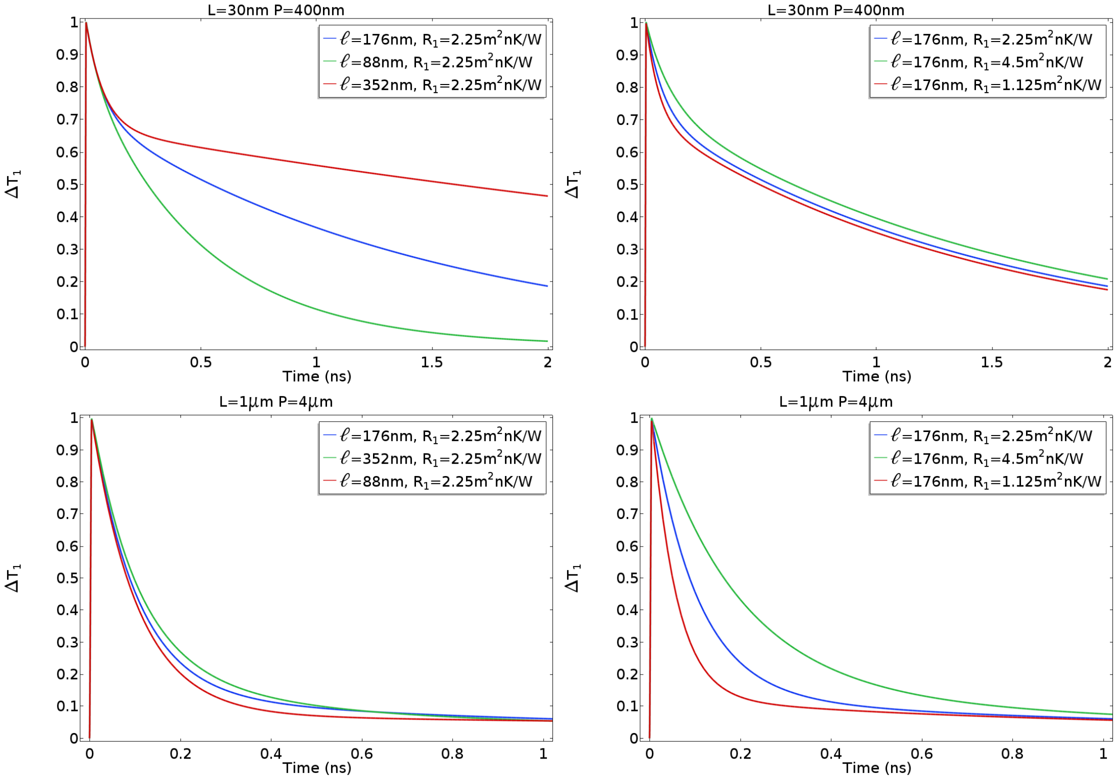

Figure 5: Sensitivity of KCM parameters. Left Plots: Thermal decay using the values given by our KCM-*ab initio* model (blue line) in comparison with the same system using a non-local length multiplied by two (green) and divided by two (red) times. Right Plots: Analogous comparison is provided but changing the used value of the resistance R_1. Top: Small heater size ($L = 30$nm and $P = 400$nm). Bottom: Large heater size ($L = 1\mu$m and $P = 4\mu$m). Notice that the boundary resistance controls the initial decay τ_1 and the non-Fourier conduction controls the decay at larger times τ_2. Moreover, the weight of the second exponential with time τ_2 increases by decreasing the line-width.

The red circles in Figure 4 identify the coefficients for large heaters with size $L = 1\mu$m ($\tau_1 = 139$ps, $\tau_2 = 1840$ps, $a_2 = 0.09$). In this sample the situation is the contrary with respect to the previous case. The hydrodynamic weight is small ($a_2 < 0.1$) and the timescale τ_1 determined by the TBR dominates. In the bottom plots of Figure 5 the decay for this sample is represented in blue, along with the same sensitivity analysis of the KCM parameters. Notice that the decay is mainly influenced by R_1 while the change of ℓ does not have much effect. This

is expected since for large heater sizes the decay is well described by Fourier's law along a Kapitza interfacial resistance.

Therefore, there is a transition from the thermal relaxation dominated by hydrodynamic effects observed for small heater sizes, to the evolution dominated by the interfacial resistance for large sizes. For intermediate sizes, the two mechanisms are important and a double exponential decay is evident (see Figure 4 of the main text). The experimental validation of this transition is displayed in Figure 6d of the main text.

The Two-Box Model: Analytical Derivation of double exponential thermal decay.

Here we derive the analytical expression for the parameters of the double exponential thermal decay predicted by KCM in the case $L < \ell$. We denote x the cross-plane direction towards the substrate and y the in-plane direction. The origin of coordinates is the center of the interface.

In KCM, the heat flux in the substrate is described through the hydrodynamic heat transport equation

$$-\kappa_{\mathrm{Si}} \nabla T = \tau \frac{\partial \vec{q}}{\partial t} + \vec{q} - \ell^2 (\nabla^2 \vec{q} + \alpha \nabla (\nabla \cdot \vec{q})) \tag{15}$$

along with the energy conservation equation

$$\nabla \cdot \vec{q} = -c_{\mathrm{Si}} \frac{\partial T}{\partial t}. \tag{16}$$

We neglect the term $\tau \frac{\partial \vec{q}}{\partial t}$ because it does not play a significant role in the present experimental conditions. We also neglect the thermo-elastic coupling in the energy conservation for simplicity. We consider the Stokes regime $L < \ell$ (heat transport dominated by viscosity); then one can neglect the term $\vec{q}$ in (15) close to the heater since we expect $\ell^2 \nabla^2 \vec{q} \sim \frac{\ell^2}{L^2} \vec{q}$. Is worth to note that the heat flux profile saturates after a fast transient and viscosity remains constant during the rest of the experiment. The heat flux profile saturation time can be estimated as $\ell^2 / \chi_{\mathrm{Si}} = 341 ps$. To illustrate this, in Figure 6 we show the time evolution of the different terms in equation (15) at $x = L$ and $y = 0$ with $L = 20$nm.

We have

$$\frac{\kappa_{\mathrm{Si}}}{\ell^2} \nabla T = \nabla^2 \vec{q} + \alpha \nabla (\nabla \cdot \vec{q}). \tag{17}$$

Now we perform integration in the region dominated by viscous effects $x < 2\ell$:

$$\frac{\kappa_{\mathrm{Si}}}{\ell^2} \int_0^{2\ell} \nabla T dx = -\frac{\kappa_{\mathrm{Si}}}{\ell^2} (T_2 - T_2^{2\ell}) = \int_0^{2\ell} dx \left(\frac{\partial^2 q_x}{\partial x^2} + \frac{\partial^2 q_x}{\partial y^2}\right) + \alpha \int_0^{2\ell} dx \frac{\partial \nabla \cdot \vec{q}}{\partial x}, \tag{18}$$

where T_2 is the substrate temperature at the interface $x = 0$ and $T_2^{2\ell}$ is the substrate temperature at $x = 2\ell$.

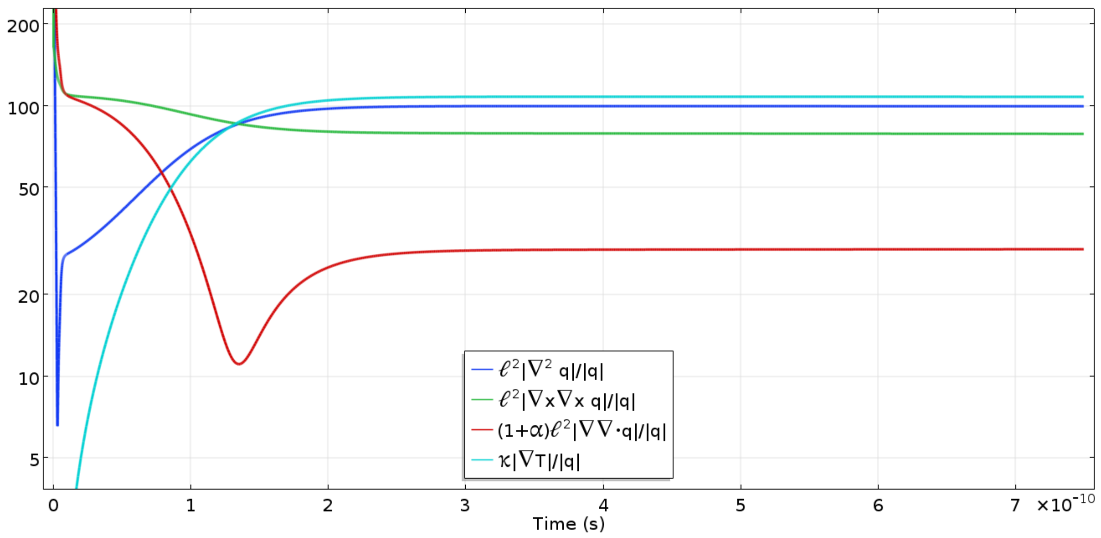

Figure 6: Time evolution of the hydrodynamic heat transport equation terms at $x = L$ and $y = 0$ with $L = 20$nm.

At the time scales considered in the experiment $T_2^{2\ell}$ is constant and close to the initial temperature $T_2^{\infty} = T_0$. Moreover, during the experimental time scale, the heat flux and its derivatives at $x = 2\ell$ are neglectable in front of the heat flux and its derivatives at the interface $x = 0$.

Therefore, we can perform integration of (16) in the hydrodynamic region to obtain

$$\int_0^{2\ell} dx \frac{\partial \nabla \cdot \vec{q}}{\partial x} = c_{\text{Si}} \frac{\partial T_2}{\partial t}, \tag{19}$$

or, equivalently,

$$\int_0^{2\ell} dx \frac{\partial^2 q_x}{\partial x^2} = c_{\text{Si}} \frac{\partial T_2}{\partial t} + \left. \frac{\partial q_y}{\partial y} \right|_{x=0}. \tag{20}$$

Introducing (19,20) in (18), we obtain

$$\frac{\kappa_{\text{Si}}}{\ell^2}(T_2 - T_2^{\infty}) + (1+\alpha) c_{\text{Si}} \frac{\partial T_2}{\partial t} = -\int_0^{2\ell} dx \frac{\partial^2 q_x}{\partial y^2} - \left. \frac{\partial q_y}{\partial y} \right|_{x=0}. \tag{21}$$

Now we average equation (21) over all the interface points $y \in [-L/2, L/2]$.

$$\frac{\kappa_{\text{Si}}}{\ell^2}(\bar{T}_2 - T_2^{\infty}) + (1+\alpha) c_{\text{Si}} \frac{\partial \bar{T}_2}{\partial t} = \frac{B}{L} \bar{q}_x \tag{22}$$

where $\bar{T}_2$, $\bar{q}_x$ are the average temperature and heat flux in the interface, respectively, and

$$B = -\frac{\int_0^{2\ell} dx \int_{-L/2}^{L/2} dy \frac{\partial^2 q_x}{\partial y^2}}{\bar{q}_x} - \frac{\left. 2q_y \right|_{y=L/2 \ \& \ x=0}}{\bar{q}_x} \tag{23}$$

is a geometric parameter related with the average heat flux profile in the hydrodynamic region. This parameter saturates because the heat flux profile reach a

stationary situation as can be seen in Figure 3. The saturated value can be estimated from the COMSOL simulations. We obtain a constant value for $L < \ell$ in the 1D geometry: $B = 3$.

Diffusion in the heater region is extremely fast and hence the temperature of the heater T_1 is uniform within the time scale of the experiment. Therefore, using the averaged form of a Kapitza interface boundary condition $\bar{q}_x = \frac{T_1 - \bar{T}_2}{R_1}$ in (22) we obtain the following evolution equation

$$\frac{\tau_S}{R_2}\frac{d\bar{T}_2}{dt} = -\frac{\bar{T}_2 - T_2^\infty}{R_2} + \frac{T_1 - \bar{T}_2}{R_1} \tag{24}$$

where

$$\tau_S = \frac{(1+\alpha)c_{\rm Si}\ell^2}{\kappa_{\rm Si}} \tag{25}$$

and

$$R_2 = \frac{B\ell^2}{\kappa_{\rm Si} L} \tag{26}$$

is the viscous resistance.

Consider now the energy conservation in the heater $c_{\rm Ni}\frac{\partial T_1}{\partial t} = -\nabla \cdot \vec{q}$. By performing volume integration of this equation with using the Kapitza interface boundary condition and the insulation condition for the other boundaries, we obtain an independent evolution equation

$$c_{\rm Ni} h \frac{dT_1}{dt} = -\frac{T_1 - \bar{T}_2}{R_1}. \tag{27}$$

Notice that, in the derivation of the evolution equations (24,27), a simplified Kapitza interface condition have been used *i.e.* the hydrodynamic contributions to the interface boundary condition (10) have been neglected for simplicity. The inclusion of the hydrodynamic contributions to the boundary condition (which cause only small deviations for extremely small $L < 50$nm) can be found in the ending notes of this section.

The heater temperature T_1 can be obtained from the system of partial differential equations (24,27):

$$T_1 - T_2^\infty = a_1 \exp(-t/\tau_1) + a_2 \exp(-t/\tau_2). \tag{28}$$

In the case of isolated heaters ($R_1 < R_2$), the system (24,27) can be simplified and we obtain

$$\tau_1 = R_1 \frac{c_{Ni} h \tau_S / R_2}{\tau_S/R_2 + c_{\rm Ni} h} \equiv R_1 \frac{C_1 C_2}{C_1 + C_2} \equiv R_1 C_{eq} \tag{29}$$

$$\tau_2 = \frac{B c_{\rm Ni} \ell^2}{\kappa_{\rm Si}} \frac{h}{L} + \frac{c_{\rm Si}\ell^2(1+\alpha)}{\kappa_{\rm Si}} = c_{\rm Ni} h R_2 + \tau_S \equiv (C_1 + C_2) R_2 \tag{30}$$

being $C_1 = c_{\rm Ni} h$, $C_2 = \tau_S/R_2$ the heat capacities of the heater and the dam region, respectively. With these definitions, the system (24,27) is equivalent to (2) in the main text.

In the case considered here ($\tau_1 < \tau_2$ *i.e.* $R_1 < R_2$),

$$a_1 = \frac{\tau_S/R_2}{c_{\text{Ni}}h + \tau_S/R_2} = \frac{C_2}{C_1 + C_2} \tag{31}$$

$$a_2 = \frac{c_{\text{Ni}}h}{c_{\text{Ni}}h + \tau_S/R_2} = \frac{C_1}{C_1 + C_2} \tag{32}$$

If $R_1 \sim R_2$, then the system (24,27) needs to be solved with no approximations. This is the case of interest for small L and $P = 4L$ (close-packed situation) in which we use a reduced non-local length ℓ_{eff} (reduced R_2). See the ending notes of this section for details.

For illustration, in Figure 7 we show the comparison between the analytical prediction (28) for the heater temperature evolution $T_1(t)$ with $L = 20nm$ and $P = 800nm$ compared with the Finite Elements calculation.

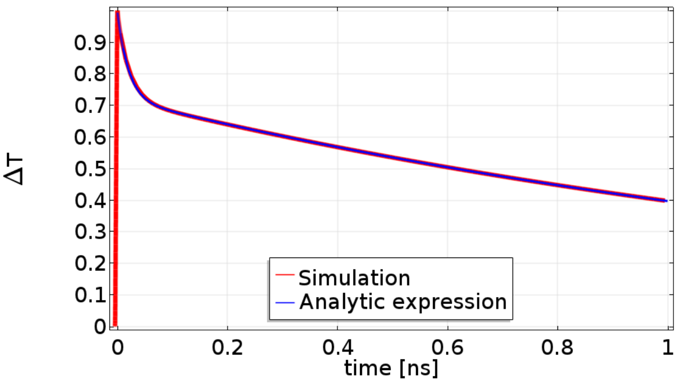

Figure 7: Expression (28) with parameters (29,30,31,32) compared with the simulated thermal decay of the heater according to KCM obtained using COMSOL Multiphysics for $L = 20nm$ and $P = 800nm$.

Note I: Solutions of the system of partial differential equations (24,27)

Here we revisit the solutions of the system of partial differential equations (24,27). Exponential solutions $\exp(tw)$ satisfy

$$w^2 + w(\frac{1}{R_1 C_{eq}} + \frac{1}{\tau_S}) + \frac{1}{\tau_S C_1 R_1} = 0 \tag{33}$$

being w the roots of the system characteristic polynomial. There are always two real negative roots $w_1 = -1/\tau_1$ and $w_2 = -1/\tau_2$. In order to find the expressions (29,30) for the characteristic times we assumed $R_2 > R_1$ and hence we simplified the previous equation to

$$w^2 + w(\frac{1}{R_1 C_{eq}}) + \frac{1}{\tau_S C_1 R_1} = 0 \tag{34}$$

Regarding the weights of the double exponential, in general we have

$$a_1 = \frac{\tau_1}{C_1 R_1} \frac{\tau_2 - C_1 R_1}{\tau_2 - \tau_1} \tag{35}$$

$$a_2 = \frac{\tau_2}{C_1 R_1} \frac{C_1 R_1 - \tau_1}{\tau_2 - \tau_1} \tag{36}$$

which in the case $\tau_1 < \tau_2$ simplify to equations (31,32).

Notice that equations (33,35,36) are the general system of equations for the parameters τ_1, τ_2, a_1, a_2. Now consider the two geometrical regimes:

(i) Isolated situation ($P - L > 2\ell$): In this case $R_2 > R_1$ so that the approximated equation (34) can be used and the explicit analytical expressions (29,30) for τ_1, τ_2 are very close to the exact solutions of the general equation (33).

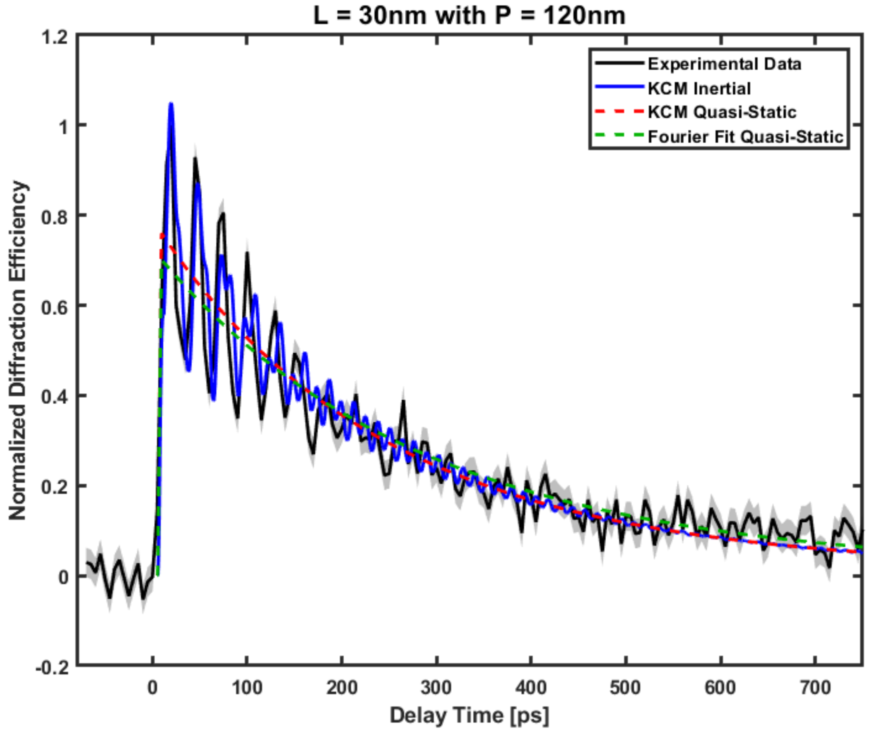

Figure 8: Comparison of the change in diffraction efficiency for experimental data, KCM, and an effective Fourier model for small heat source size and spacing (close-packed). The experimental change in diffraction efficiency for $L = 30$nm with $P = 120$nm is shown in black and error is embodied in grey shading. The KCM prediction is shown in blue including the full inertial calculations (solid) and the quasi-static (dashed). An effective Fourier model with a fitted thermal boundary resistance is shown in green. As predicted by the two-box model, we recover a Fourier-like behavior in this extreme close-packed situation.

(ii) Close-packed situation ($P = 4L$ and small L): In this case $\ell_{\text{eff}} = (P - L)/2$ so that $R_1 \sim R_2$. In this case the use of the approximated equation (34) is not

acceptable and hence we use the exact solutions of the general equation (33) to compare with experiments. It is easy to show that by reducing L with $P = 4L$, a_2 goes to 1 and a_1 goes to 0; τ_2 goes to the Fourier decay time $\tau_F = c_{\mathrm{Ni}} h R_1$ and τ_1 tends to zero. Therefore, we recover Fourier in this limit and we don't expect a clearly observable double exponential decay. In particular, for $L < 50\mathrm{nm}$, $P = 4L$ we expect a single exponential decay as in the Fourier-based description. This limit is consistent with experimental observations as shown in Figure 8.

Note II: Inclusion of the hydrodynamic contributions to the interface boundary condition (10) in the two box model.

Consider the hydrodynamic interface boundary condition (10) instead of the simplified Kapitza condition. We average it along the interface with expressing $\nabla \cdot \vec{q}$ and $\nabla \vec{q}$ as quantities proportional to the interface normal heat flux $\bar{q_x}$ (this is possible because of the saturation of the heat flux profile close to the interface):

$$(T_1 - \bar{T_2}) = \bar{q_x}[R_1 - \frac{1}{\gamma^{\mathrm{Si}} L}(\beta b_1 - \chi_{xx} b_2 - \chi_{yy} b_3)] \tag{37}$$

where

$$b_1 = \frac{\int_{-L/2}^{L/2} dy \nabla \cdot \vec{q}}{\bar{q_x}} = 1.5 \tag{38}$$

$$b_2 = \frac{\int_{-L/2}^{L/2} dy \frac{\partial q_x}{\partial x}}{\bar{q_x}} = -1 \tag{39}$$

$$b_3 = \frac{\int_{-L/2}^{L/2} dy \frac{\partial q_y}{\partial y}}{\bar{q_x}} = 2.5 \tag{40}$$

are dimensionless parameters estimated from the corresponding saturated value in the COMSOL simulations.

Now we can input this averaged boundary condition to the $\bar{T_2}$ evolution equation (22) and the heater energy conservation equation to obtain the two box model system of equations. This system is exactly the same as the one previously obtained (24,27) with a redefined interface boundary resistance R_1':

$$R_1' = R_1 + \frac{1}{\gamma^{\mathrm{Si}} L}(-\beta b_1 + \chi_{xx} b_2 + \chi_{yy} b_3). \tag{41}$$

Therefore, by including the hydrodynamic contributions to the boundary condition we obtain a larger thermal resistance which become explicitly geometry dependent. This correction slightly increase the τ_1 value for small sizes reported in Figure 6 of the main text with respect to the value obtained using the simplified Kapitza boundary resistance. However, for $L > 50\mathrm{nm}$, we have $R_1 \sim R_1'$.

3 Matrix Pencil Method

We utilize the matrix pencil method (MPM) to filter the elastic wave oscillations out of the experimental change in diffraction efficiency signal. Our MPM algorithm begins by forming the time-lag co-variance matrix from an experimental time signal (*i.e.* the diffraction efficiency signal as a function of pump-probe time delay). We then perform the singular value decomposition of this matrix and create a scree plot of the singular values. From this plot, we separate the relevant time-correlated patterns in the data from the random noise. Next, we compute the complex exponential ($e^{(\alpha+i\beta)t}$) that best represents each relevant component. The result is a decomposition of the experimental signal into a few complex exponentials and random noise shown in Figure 9. We remove the oscillatory exponentials (exponentials with complex arguments) since they are not of interest in this study, then sum the remaining exponentials and noise. The final result is an experimental time signal without oscillations from elastic waves also shown in Figure 9. In essence, MPM is similar to a robust least squares fitting of multiple complex exponentials to the experimental data. In the presence of noise, MPM can more precisely and accurately extract the damped oscillations than a Fourier transform. A simple Fourier transform (which assumes stationary oscillations) is a sufficient choice for higher signal-to-noise, long lived periodic signals. A simple example is shown in Figure 10. Mathematical details of MPM along with validation and comparison to a Fourier transform can be found in [15, 16, 17]. An example application can be found in [18] and the numerical algorithm used in this work can be found in [19].

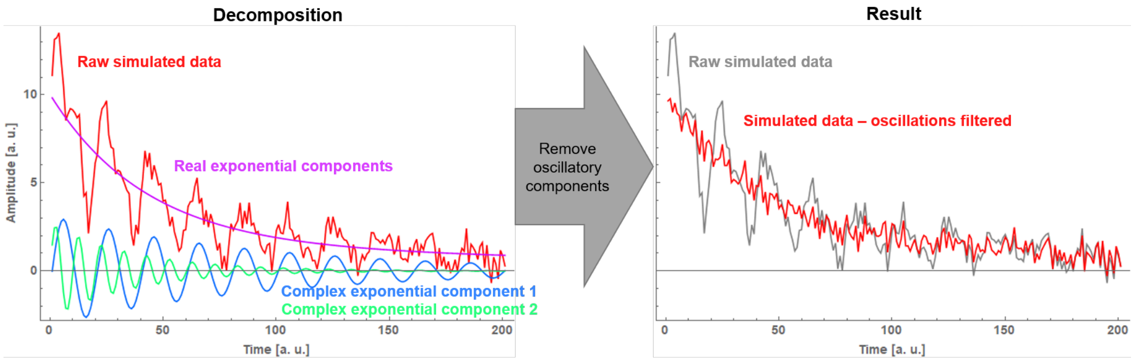

Figure 9: Matrix pencil method (MPM) is used to remove the oscillations of the elastic waves from the experimental data. Left is the MPM decomposition of simulated data with gaussian noise. MPM breaks down the signal into exponentials with real arguments, exponentials with complex arguments, and noise. Right is the re-constructed signal without the oscillatory (exponentials with complex arguments) components.

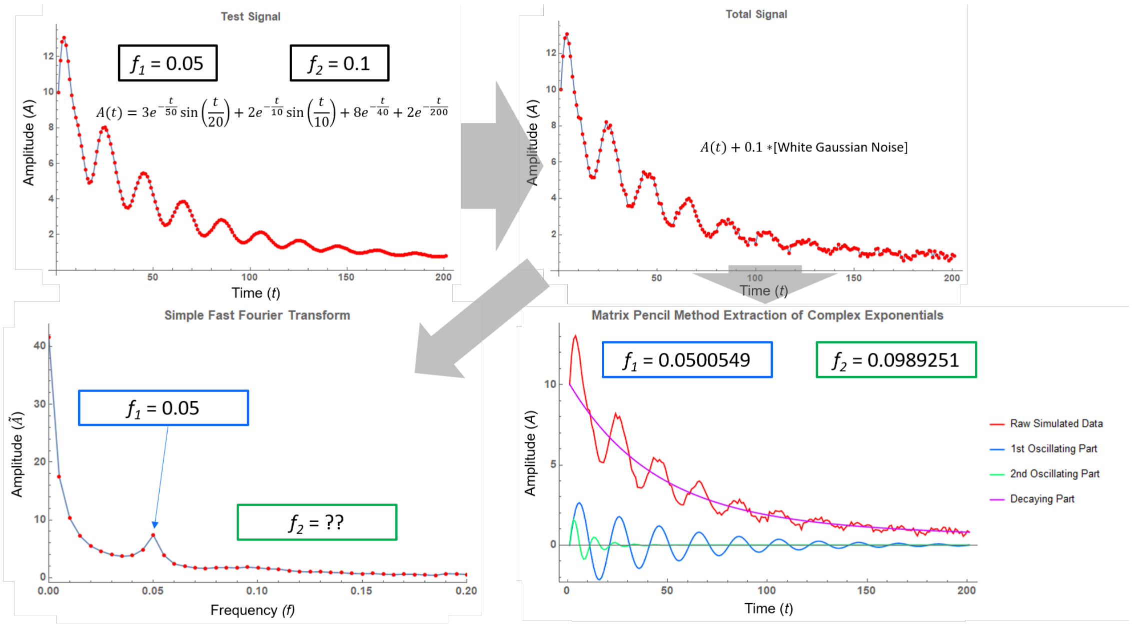

Figure 10: Top left shows a test signal generated by the addition of two damped sinusoids and two exponential decays (a good approximation of our experimental signal). Top right shows a small amount of white Gaussian noise added to the test signal. Bottom right shows that matrix pencil method (MPM) decomposition of the signal which identifies two oscillations. MPM extracts not only frequency values very close to the original test signal but also the decay times of each oscillation. Bottom left shows that a simple fast Fourier transform (FFT) identifies—with high accuracy—the obvious frequency but that highly damped oscillation is hidden by the noise. These results highlight the comparison of MPM and a simple FFT.

4 Atomic Force Microscopy

We perform atomic force microscopy (AFM) measurements to characterize the sizes and periods of the nanostructured transducers arrays (nano-gratings) utilized in the experiment. As mentioned in *Methods*, we fabricate metallic nickel nanostructures on the surface of a silicon substrate using e-beam lithography techniques. We characterize the linewidth L, periodicity P, and height h of the nano-gratings for both nanoline (1D-confined) and nanodot (2D-confined) geometries. An example AFM measurement is shown in Figure 11.

We calculate L by measuring the width at the top of the nanostructure in the AFM image, P by measuring the distance between peaks in the auto-correlation of the AFM image, and h by computing the difference between the two peaks which appear in a histogram of the heights in the AFM image (one peak corresponds to the substrate while the other corresponds to the top of the nanostructures). The results of these measurements, see Table 2, confirm the high quality of the fabrication. We use these values in the numerical KCM simulations. Because the fabrication technique creates a uniform height of nanostructures across the entire sample, we use the same average height value for all nano-gratings.

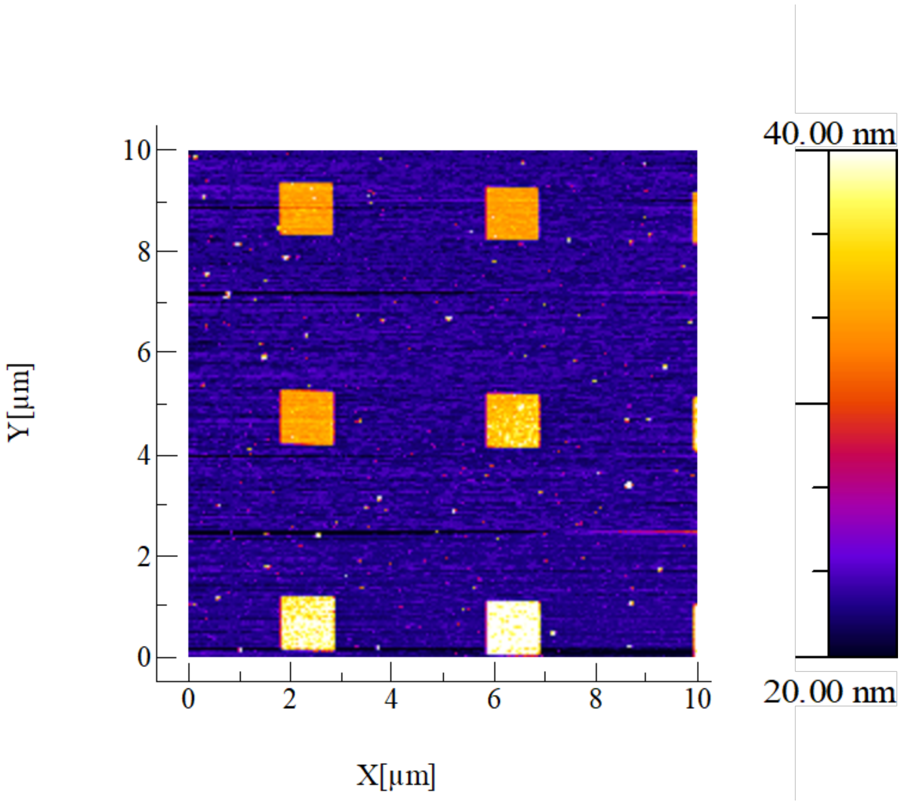

Figure 11: Example atomic force microscopy (AFM) image for a nominal $L = 1000nm$ and $P = 4000nm$ nanodot array

For any nano-grating geometries with no L or P AFM values in Table 2, we use nominal L and P with the average value for h in the numerical KCM simulations.

5 More Details on Extraction of Decay Times

In Figure 6 of the article, the agreement between numerical predictions and experimental measurements for τ_1 and τ_2 is quite good. However, there exists disagreement between numeric simulations and experiments for the interface decay (τ_1) for small linewidth L and for the hydrodynamic decay (τ_2) for large linewidth L. For small L, the amplitude of the interface decay (τ_1) is much smaller than the amplitude of the hydrodynamic decay (τ_2). Thus, the experimental extraction of τ_1 becomes more challenging. For large L, the interface decay dominates over the hydrodynamic decay making it more difficult to experimentally observe the hydrodynamic decay.

When extracting τ_1 from the experimental measurements for small L, we encounter additional challenges since not only is the amplitude of the τ_1 exponential small but also τ_1 is small. When τ_1 is small, it is on a similar scale as the finite response time of the nanostructures and an elastic wave oscillation. Although the pump laser pulse has an ultrashort duration (≈ 25fs), the deformation of the

Nanoline arrays				Nanodot arrays			
Nominal L,P [nm]	AFM L [nm]	AFM P [nm]	AFM h [nm]	Nominal L,P [nm]	AFM L [nm]	AFM P [nm]	AFM h [nm]
1000,4000	950±40	4050±40	11.5±1	1000,4000	1000±20	4080±40	11.5±1
300,1200	260±10	1240±10	11.5±1	300,1200	300±20	1220±20	11.5±1
200,800	-	-	11.5±1	50,400	51±4	406±4	11.5±1
100,400	-	-	11.5±1	50,200	54±5	200±5	11.5±1
50,400	51±3	405±4	11.5±1	30,120	-	-	11.5±1
50,200	49±2	205±4	11.5±1	-	-	-	-
30,400	35±3	407±3	11.5±1	-	-	-	-
30,120	30±3	122±2	11.5±1	-	-	-	-
20,400	29±4	406±5	11.5±1	-	-	-	-
20,80	-	-	11.5±1	-	-	-	-

Table 2: Table of compiled AFM measurements of nano-grating geometries. L is linewidth, P is period, and h is height. Uncertainty is calculated by standard deviation of measurements and AFM image pixel size. We use WSxM to analyze the raw AFM images [20].

system responds on the time scale of 10ps (we measure surface deformation with the probe beam). These inertial effects can inhibit our ability to extract τ_1

It is challenging to extract τ_1 for small L, close-packed (small period P) nanostructure arrays. In the extreme case where $L = 20\mathrm{nm}$ in the close-packed regime, the predicted $\tau_1 \approx 10\mathrm{ps}$ while the experimentally observed time required for the nanostructure to reach maximum thermal expansion is $\approx 15\mathrm{ps}$. Therefore, we cannot extract τ_1 for very small, close-packed nanostructure arrays. For slightly larger L, such as $L = 60\mathrm{nm}$, we can extract τ_1; however, the oscillations from elastic waves, the finite response time of the surface deformation, and the chosen experimental time sampling can artificially increase the extracted value of τ_1. In the example shown in Figure 12, the τ_1 extracted from experimental data is much different than that predicted by quasi-static numerical simulations. However, we also find deviations in the value extracted from inertial numerical simulations compared to the original value predicted by numerical quasi-static simulation, even though the both simulations are in agreement as shown in the article. Since τ_1 is on the similar time scale as one elastic wave oscillation, we cannot extract τ_1 with high accuracy. In Figure 12, sampling the numerical inertial simulation to match the experimental time resolution and then filtering the elastic wave oscillations does not result in a more accurate value.This example suggests that inertial effects, *e.g.* elastic waves, can inhibit the ability to observe τ_1 in this regime.

It is also challenging to extract τ_1 for small L, effectively isolated (large period P) nanostructure arrays. Because the change in diffraction efficiency signal is dominated by nanostructure expansion at small L [21], the signal-to-noise ratio is related to the duty cycle, *i.e.* the ratio L/P. For small L and large P, the signal-to-noise ratio is low and thus extraction of τ_1 is challenging. As shown

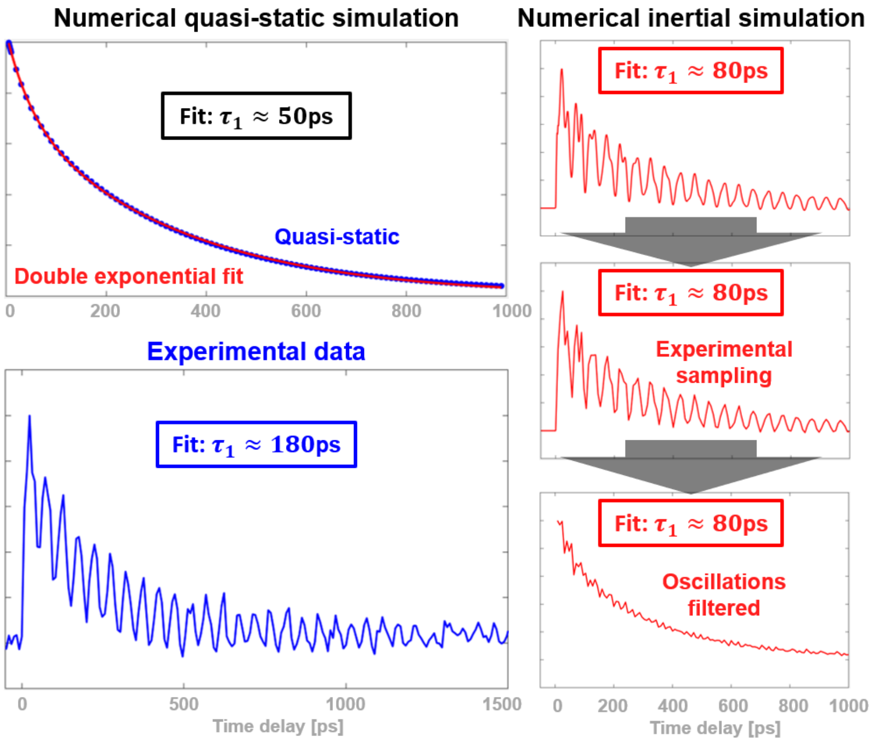

Figure 12: Example of inertial effects inhibiting observation of τ_1 where linewidth $L = 60\text{nm}$ and period $P = 240\text{nm}$. Extracting τ_1 from numerical quasi-static simulations results in a value of $\approx 80\text{ps}$ as seen in upper left. This τ_1 value is much different than what we experimentally observe, shown in the lower left. However, the values of τ_1 extracted from a numerical inertial simulation are also larger than predicted by quasi-static simulations, shown in the top right. This example suggests that inertial effects, *e.g.* elastic waves, can inhibit the ability to observe τ_1 in this regime. With data sampled at the experimental resolution, filtering the acoustic oscillations does not result in a more accurate value for τ_1 shown in bottom right.

in Figure 13, the τ_1 extracted from experimental data is almost 4 times higher than that predicted by quasi-static numerical simulations. The extraction of τ_1 from the numerical inertial simulation is about 30% higher than the quasi-static prediction. This discrepancy between the two simulations is most likely due to inertial effects as previously described. However, if we sample the inertial simulation at the experimental time resolution, the extracted τ_1 is now three times higher than the quasi-static prediction. Although our experimental capabilities allow us to capture $< 10\text{fs}$ dynamics, observing the entire thermal decay ($\sim \text{ns}$) with fs resolution creates massive experimental data sets. Therefore, we chose the experimental time sampling to be on the order of 10ps, even though it is far from technique limits. If we add Gaussian noise (same signal-to-noise ratio as experiment) to the inertial simulation sampled at experimental resolution, our fit algorithm cannot obtain reasonable values for a fitted τ_1. These results suggests that inertial effects, sampling, and noise inhibit our ability to accurately

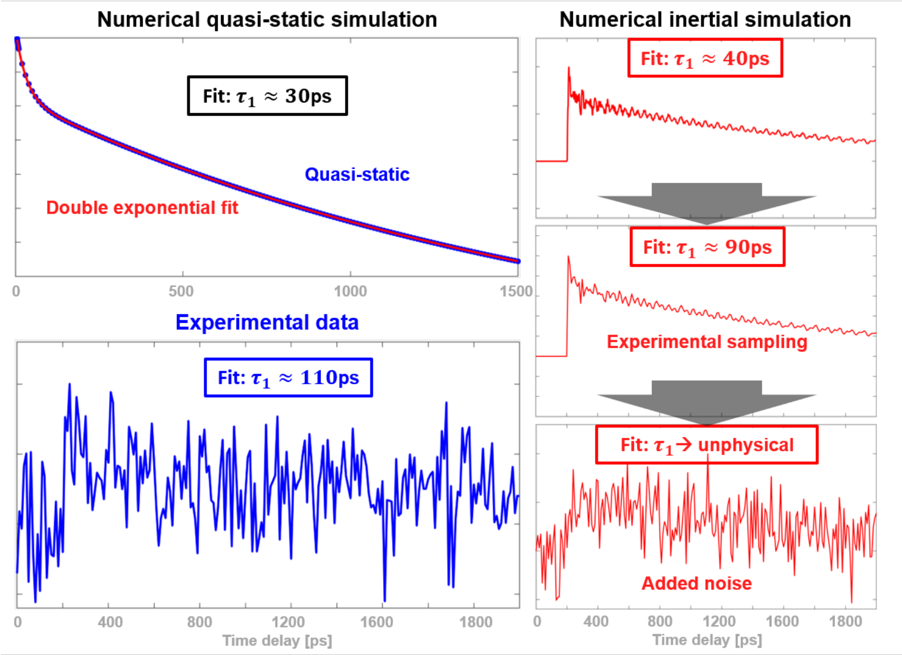

Figure 13: Example of inertial effects, sampling, and noise inhibiting observation of τ_1 where linewidth $L = 20\text{nm}$ and period $P = 400\text{nm}$. The value of τ_1 extracted from experimental data (bottom left) is almost 4 times higher than that predicted by numerical quasi-static simulations (top left). The extracted values of τ_1 from the numerical inertial simulations is about 30% higher than the quasi-static prediction (top right). However, if we sample the inertial simulation at the experimental time resolution and add Gaussian noise, we cannot extract a reasonable value for τ_1 (bottom right). Experimental data is from [21]

extract τ_1 for extremely small L. Note, that the error bars in Figure 6 in the article are computed from the standard deviation of the extracted τ_1 values; therefore, the error bars embody the precision of the experimental data and not necessarily the accuracy of the fit procedure. Note that the quasi-static predictions shown in Figures 12 and 13 use different intrinsic parameters than the results in the article.

References

[1] L.D. Landau and E.M. Lifshitz. “ Theory of Elasticity: Vol. 7 of Course of Theoretical Physics ”. In: *Pergamon Press,* Oxford, United Kingdom (1970).

[2] R Zhu, E Pan, P W Chung, X Cai, K M Liew, and A Buldum. “Atomistic Calculation of Elastic Moduli in Strained Silicon”. In: *Semiconductor Science and Technology* 21.7 (June 2006), pp. 906–911.

[3] Damiano Nardi, Marco Travagliati, Mark E. Siemens, Qing Li, Margaret M. Murnane, Henry C. Kapteyn, Gabriele Ferrini, Fulvio Parmigiani, and Francesco Banfi. “Probing Thermomechanics at the Nanoscale: Impulsively

Excited Pseudosurface Acoustic Waves in Hypersonic Phononic Crystals". In: *Nano Letters* 11.10 (2011), pp. 4126–4133.

[4] M. I. Kaganov, I. M. Lifshitz, and L. V. Tanatarov. "Relaxation between Electrons and the Crystalline Lattice". In: *Soviet Physics Journal of Experimental and Theoretical Physics* 4 (1957), pp. 173–178.

[5] F. Banfi, F. Pressacco, B. Revaz, C. Giannetti, D. Nardi, G. Ferrini, and F. Parmigiani. "*Ab Initio* Thermodynamics Calculation of All-Optical Time-Resolved Calorimetry of Nanosize Systems: Evidence of Nanosecond Decoupling of Electron and Phonon Temperatures". In: *Physical Review B - Condensed Matter and Materials Physics* 81 (2010), pp. 1–5. ISSN: 10980121.

[6] Yangyu Guo and Moran Wang. "Phonon Hydrodynamics for Nanoscale Heat Transport at Ordinary Temperatures". In: *Physical Review B* 97.3 (2018). ISSN: 24699969.

[7] R. A. Guyer and J. A. Krumhansl. "Solution of the Linearized Phonon Boltzmann Equation". In: *Physical Review* 148.2 (Aug. 1966), pp. 766–778. ISSN: 0031-899X.

[8] Amirkoushyar Ziabari, Pol Torres, Bjorn Vermeersch, Yi Xuan, Xavier Cartoixà, Alvar Torelló, Je-Hyeong Bahk, Yee Rui Koh, Maryam Parsa, Peide D. Ye, F. Xavier Alvarez, and Ali Shakouri. "Full-Field Thermal Imaging of Quasiballistic Crosstalk Reduction in Nanoscale Devices". In: *Nature Communications* 9.1 (Dec. 2018), p. 255. ISSN: 2041-1723.

[9] P. Torres, A. Ziabari, A. Torelló, J. Bafaluy, J. Camacho, X. Cartoixà, A. Shakouri, and F. X. Alvarez. "Emergence of Hydrodynamic Heat Transport in Semiconductors at the Nanoscale". In: *Physical Review Materials* 2.7 (2018), p. 076001. ISSN: 2475-9953.

[10] A. Beardo, M. Calvo-Schwarzwälder, J. Camacho, T.G. Myers, P. Torres, L. Sendra, F.X. Alvarez, and J. Bafaluy. "Hydrodynamic Heat Transport in Compact and Holey Silicon Thin Films". In: *Phys. Rev. Applied* 11 (3 Mar. 2019), p. 034003.

[11] A. Beardo, M. G. Hennessy, L. Sendra, J. Camacho, T. G. Myers, J. Bafaluy, and F. X. Alvarez. "Phonon hydrodynamics in Frequency-Domain Thermoreflectance Experiments". In: *Phys. Rev. B* 101 (7 Feb. 2020), p. 075303.

[12] P. Torres, A. Torelló, J. Bafaluy, J. Camacho, X. Cartoixà, and F. X. Alvarez. "First Principles Kinetic-Collective Thermal Conductivity of Semiconductors". In: *Physical Review B* 95.16 (Apr. 2017), p. 165407. ISSN: 2469-9950.

[13] F X Alvarez, D Jou, and A Sellitto. "Phonon hydrodynamics and Phonon-Boundary Scattering in Nanosystems". In: *Journal of Applied Physics* 105.1 (2009), p. 14317.

[14] Yongjie Hu, Lingping Zeng, Austin J. Minnich, Mildred S. Dresselhaus, and Gang Chen. "Spectral Mapping of Thermal Conductivity through Nanoscale Ballistic Transport". In: *Nature Nanotechnology* 10.8 (June 2015), pp. 701–706. ISSN: 1748-3387.

[15] Y. Hua and T. K. Sarkar. "Matrix Pencil Method to Estimate the Parameters of Exponentially Damped/Undamped Sinusoids in Noise". In: *IEEE Trans. Acoust., Speeh, Signal Process.* 38 (1990), pp. 814–824.

[16] T. K. Sarkar and O. Pereira. "Using the Matrix Pencil Method to Estimate the Parameters of a Sum of Complex Exponentials". In: *IEEE Antennas and Propagation Mag.* 37 (1995), pp. 48–55.

[17] J. E. Fernandez del Rio and T. K. Sarkar. "Comparison between the Matrix Pencil Method and the Fourier Transform Technique for High-Resolution Spectral Estimation". In: *Digit. Signal Process.* 6 (1996), pp. 108–125.

[18] K. M. Hoogeboom-Pot, E. Turgut, J. N. Hernandez-Charpak, J. M. Shaw, H. C. Kapteyn, M. M. Murnane, and D. Nardi. "Nondestructive Measurement of the Evolution of Layer-Specific Mechanical Properties in sub-10 nm Bilayer Films". In: *Nano Letters* 16 (2016), pp. 4773–4778.

[19] K. M. Hoogeboom-Pot. "Uncovering New Thermal and Mechanical Behavior at the Nanoscale Using Coherent Exterme Ultraviolet Light". PhD thesis. CU Boulder, 2015.

[20] I. Horcas, R. Fernández, J. M. Gómez-Rodríguez, J. Colchero, J. Gómez-Herrero, and A. M. Baro. "WSXM: A Software for Scanning Probe Microscopy and a Tool for Nanotechnology". In: *Rev. Sci. Instrum.* 78 (2007), p. 013705.

[21] Travis D. Frazer, Joshua L. Knobloch, Kathleen M. Hoogeboom-Pot, Damiano Nardi, Weilun Chao, Roger W. Falcone, Margaret M. Murnane, Henry C. Kapteyn, and Jorge N. Hernandez-Charpak. "Engineering Nanoscale Thermal Transport: Size- and Spacing-Dependent Cooling of Nanostructures". In: *Phys. Rev. Applied* 11 (2 Feb. 2019), p. 024042.
